# EZH2 Inhibition Enhances PD‐L1 Protein Stability Through USP22‐Mediated Deubiquitination in Colorectal Cancer

**DOI:** 10.1002/advs.202308045

**Published:** 2024-03-22

**Authors:** Jiaqi Huang, Qianqian Yin, Yuqing Wang, Xin Zhou, Yunyun Guo, Yuanjun Tang, Rui Cheng, Xiaotong Yu, Jie Zhang, Chen Huang, Zhanya Huang, Jianlin Zhang, Zhengyang Guo, Xiao Huo, Yan Sun, Yanfang Li, Hao Wang, Jianling Yang, Lixiang Xue

**Affiliations:** ^1^ Peking University Third Hospital Cancer Center Department of Radiation Oncology Peking University Third Hospital Beijing 100191 China; ^2^ Center of Basic Medical Research Institute of Medical Innovation and Research Peking University Third Hospital Beijing 100191 China; ^3^ Biobank Peking University Third Hospital Beijing 100191 China; ^4^ Cancer Center of Peking University Third Hospital Beijing 100191 China; ^5^ Department of General Surgery Peking University Third Hospital Beijing 100191 China; ^6^ Medicine Innovation Center for Fundamental Research on Major Immunology‐related Diseases Beijing 100191 China

**Keywords:** colon cancer, deubiquitinase, epigenetics, EZH2, PD‐L1, tumor immunity

## Abstract

The regulation of PD‐L1 is the key question, which largely determines the outcome of the immune checkpoint inhibitors (ICIs) based therapy. However, besides the transcription level, the protein stability of PD‐L1 is closely correlated with its function and has drawn increasing attention. In this study, EZH2 inhibition enhances PD‐L1 expression and protein stability, and the deubiquitinase ubiquitin‐specific peptidase 22 (USP22) is identified as a key mediator in this process. EZH2 inhibition transcriptionally upregulates USP22 expression, and upregulated USP22 further stabilizes PD‐L1. Importantly, a combination of EZH2 inhibitors with anti‐PD‐1 immune checkpoint blockade therapy improves the tumor microenvironment, enhances sensitivity to immunotherapy, and exerts synergistic anticancer effects. In addition, knocking down USP22 can potentially enhance the therapeutic efficacy of EZH2 inhibitors on colon cancer. These findings unveil the novel role of EZH2 inhibitors in tumor immune evasion by upregulating PD‐L1, and this drawback can be compensated by combining ICI immunotherapy. Therefore, these findings provide valuable insights into the EZH2‐USP22‐PD‐L1 regulatory axis, shedding light on the optimization of combining both immune checkpoint blockade and EZH2 inhibitor‐based epigenetic therapies to achieve more efficacies and accuracy in cancer treatment.

## Graphical abstract

Inhibitors of EZH2 can activate the expression of deubiquitinase USP22 in colorectal cancer via canonical epigenetic regulation through H3K27me3, thereby stabilizing PD‐L1 protein and enhancing its expression, inducing immune evasion and diminishing the anti‐tumor therapeutic efficacy. Combination immunotherapy can block the immune suppression effects of EZH2 inhibitors and exert a combined anti‐cancer effect.

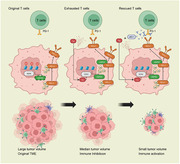

## 1 Introduction

With a deeper understanding of cancer, immune‐related characteristics, including avoiding immune destruction and tumor‐promoting inflammation, have been increasingly regarded as hallmarks of cancer.^[^ ^1^ ^]^ Immunotherapies, especially immune checkpoint inhibitors (ICIs) based on monoclonal antibodies represented by anti‐PD‐1/PD‐L1 antibodies,^[^ ^2^ ^]^ are the most promising therapeutic strategies to change the landscape of tumor treatment.^[^ ^3^ ^]^ However, only 20–40% of patients respond to ICIs, and even fewer will have long‐term disease remission.^[^ ^4^, ^5^, ^6^, ^7^ ^]^ Various indicators, including PD‐L1 (including exo PD‐L1), TMB, high microsatellite instability (MSI‐H), and mismatch repair‐deficient (dMMR), are considered capable of predicting populations who would benefit from immunotherapy. Generally, the level of PD‐L1 expression is widely considered as a potential biomarker of anti‐PD‐1/PD‐L1 immunotherapy.^[^ ^8^, ^9^ ^]^

Colorectal cancer (CRC) is the second leading cause of cancer‐related death worldwide,^[^ ^10^ ^]^ and immunotherapy has been used for colorectal cancer with MSI‐H or dMMR.^[^ ^11^ ^]^ Although patients with high PD‐L1 expression may benefit from immunotherapy,^[^ ^12^ ^]^ the dynamic expression of PD‐L1 affected by treatments remains unclear. PD‐L1 expression can be regulated by factors including transcriptional, post‐ transcriptional, and translational levels, and increasing attention is being given to the importance of post‐translational regulation of PD‐L1.^[^ ^13^ ^]^ Therefore, unraveling the regulatory mechanisms underlying PD‐L1 expression could further facilitate the identification of populations sensitive to immunotherapy or provide novel targets for combined treatment strategies.

Nonmutational epigenetic reprogramming has been identified as a new hallmark of cancers. which also participates in the regulation of PD‐L1 expression.^[^ ^14^, ^15^, ^16^, ^17^, ^18^ ^]^ The histone methyltransferase enhancer of zeste homolog 2 (EZH2), the catalytic subunit of polycomb repressive complex 2 (PRC2), is involved in promoting tumor development, proliferation, invasion, metabolism, and antitumor immunity.^[^ ^19^, ^20^, ^21^, ^22^ ^]^ Although EZH2 participates in regulating PD‐L1 expression,^[^ ^15^, ^16^ ^]^ it is not clear whether EZH2 affects the modification of PD‐L1 protein levels.

Our study suggests a novel regulatory mechanism of EZH2 in manipulating PD‐L1 expression through post‐translational modifications. Intriguingly, we identify the deubiquitinase USP22 as a potential key player in EZH2‐mediated alteration of PD‐L1 protein stability. These findings provide valuable insights into the intricate interplay between EZH2 and PD‐L1, shedding light on the optimization of combining both immune checkpoint blockade and EZH2 inhibitor‐based epigenetic therapies to achieve more efficacy.

## 2 Results

### 2.1 EZH2 Inhibition Upregulates PD‐L1 Expression in Colorectal Cancers

**Figure 1:**
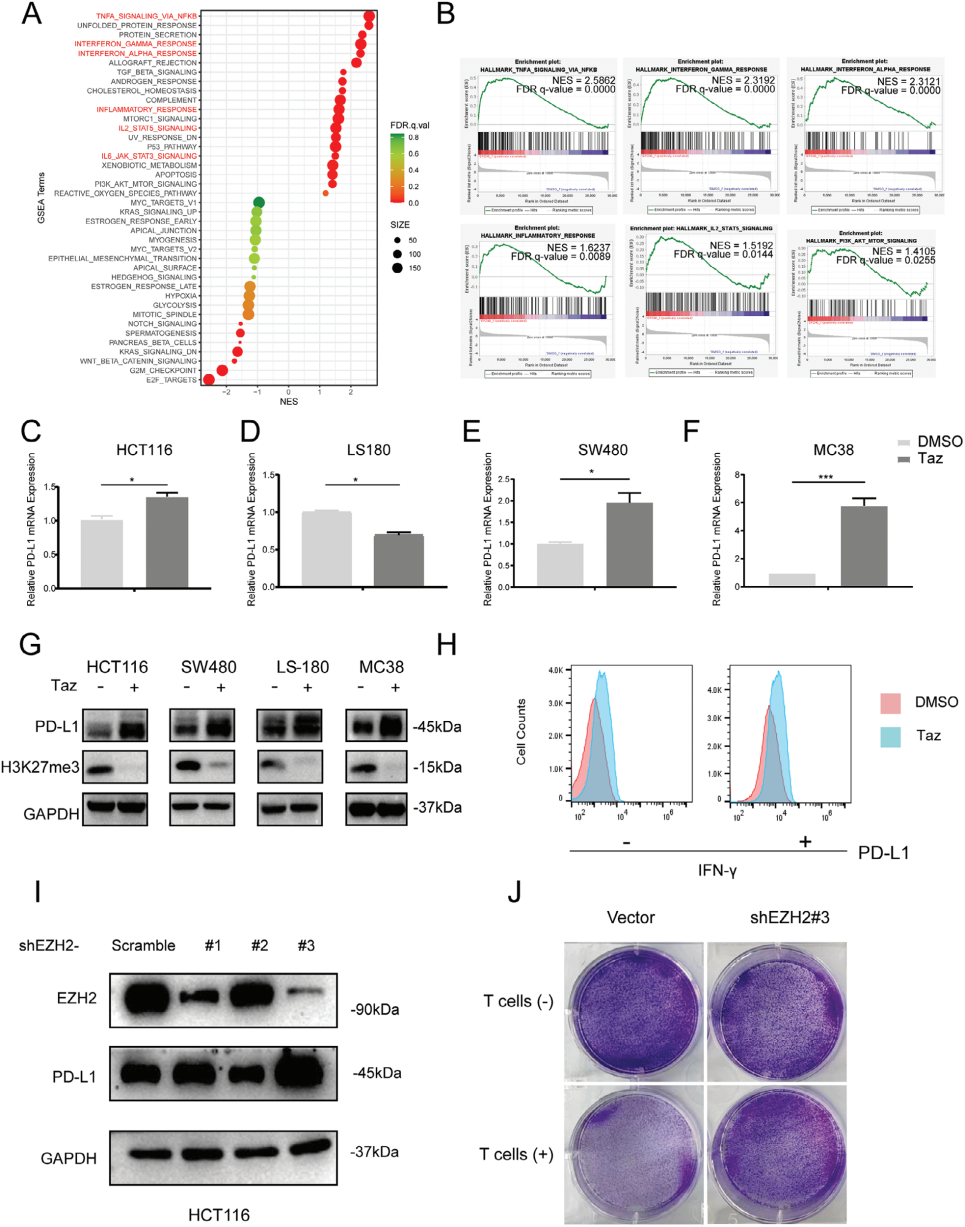
EZH2 inhibition enhance the expression of PD‐L1 in colorectal cancer. A) Gene set enrichment analysis in HCT116 cells treated with Taz 40 µM comparing to DMSO control. HCT116 cells were treated with 40 ng mL^−1^ IFN‐γ for an additional 24 h. Immune‐related gene sets enriched in Taz‐treated tumor cells are labeled in red. B) GSEA analysis highlighting six Taz‐induced immune relate enriched gene sets. C–F) Representative qRT‐PCR analysis of PD‐L1 mRNA level in various colon cancer cell lines, C)HCT116, D)LS180, E)SW480, and F)MC38 cells were treated with Taz for 48 h and 40 ng mL^−1^ IFN‐γ for additional 24 h. mean ± SEM, two‐tailed unpaired *t*‐test. G) Representative immunoblot analysis of various colon cancer cell lines treated with Taz for 48 h and 40 ng mL^−1^ IFN‐γ for an additional 24 h. H) Flow cytometry assay showing cell surface expression of PD‐L1 after treatment by Taz with or without the presence of IFN‐γ. Experiments were repeated twice independently with similar results. I) Representative immunoblot analysis of the effects of EZH2 knockdown on PD‐L1 expression in HCT116 cells after treatment with EZH2 shRNAs or scrambled shRNA. 40 ng mL^−1^ IFN‐γ was added for an additional 24 h. J) HCT116 cell survival upon incubation with activated *T*‐cells. HCT116 cells were cocultured with or without activated *T*‐cells at a ratio of 1:5 for 72 h and subjected to crystal violet staining. ns., not significant; *p > *0.05, ^*^ *p < *0.05, ^**^ *p < *0.01, ^***^ *p < *0.001.

To validated the inhibitory effects of EZH2 inhibition, HCT116 colon cancer cells were treated with Tazemetostat (Taz, also known as EPZ‐6438), an FDA‐approved EZH2 inhibitor (EZH2i). Taz inhibits cell proliferation and viability in a dose‐dependent manner (Figure S1A,B, Supporting Information) in vitro. To further reveal the global impacts of EZH2 functional suppression on colon tumor cells, we conducted transcriptome analysis of HCT116 colon cancer cells treated with Taz,. Gene set enrichment analysis (GSEA) showed that immune‐related signaling pathways were enriched in EZH2i‐treated HCT116 cells (**Figure** **1**A,B), such as the interferon response and inflammatory response, which are known to be associated with PD‐L1 expression and the suppression of host immune function.^[^ ^23^, ^24^, ^25^ ^]^ Furthermore, we found that low expression of EZH2 also has an activating effect on these immune‐related pathways through reanalysis of colorectal cancer single‐cell sequencing data(Figure S1C, Supporting Information).^[^ ^26^ ^]^ However, despite Taz activating immune‐related pathways, only mild effects of PD‐L1 at the mRNA level were observed after Taz treatment in various colon cancer cell lines (Figure 1C–F). Yet, Taz treatment exerts a more pronounced upregulation effect on PD‐L1 protein in colon cancers (Figure 1G). Flow cytometry analysis also confirmed that Taz treatment can upregulate the expression of PD‐L1 on the cell surface (Figure 1H). Moreover, EZH2 knockdown by shRNA resulted in an upregulation of PD‐L1 (Figure 1I). Coculture experiment further indicated that inhibiting EZH2 function significantly enhanced resistance to *T*‐cell‐mediated cytotoxicity on tumor cells (Figure 1J; Figure S1D, Supporting Information). These findings indicate that EZH2 might regulate PD‐L1 expression through post‐translational modifications rather than solely at the transcriptional level,^[^ ^15^ ^]^ further suggesting a potential role for EZH2 in modulating post‐translational regulation of PD‐L1 to evade immune surveillance.

### 2.2 EZH2 Regulates the Stability of the PD‐L1 Protein by Modulating K48‐Mediated Ubiquitination

**Figure 2:**
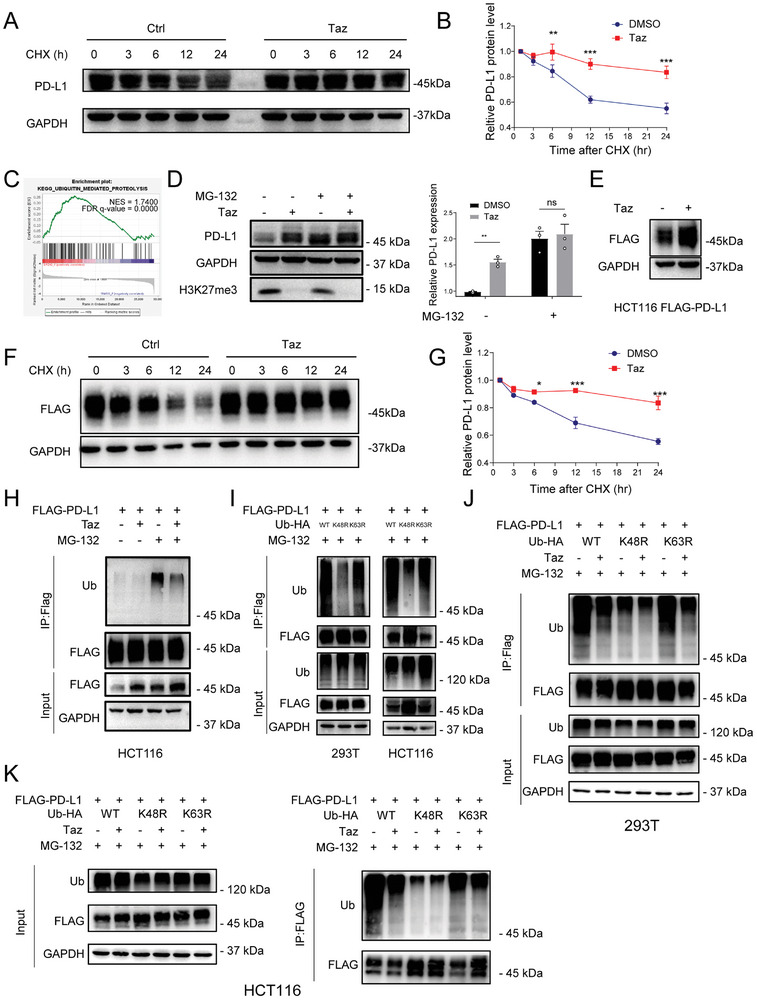
Inhibition of EZH2 enhances the stability of PD‐L1 protein and attenuates K48‐mediated ubiquitination. A) Representative immunoblot and B) quantification analysis of endogenous PD‐L1 degradation in HCT116 treated with Taz or DMSO control, 20 µg mL^−1^ cycloheximide (CHX) was incubated indicated time. Results are presented as mean ± SEM, two‐tailed unpaired *t*‐test. C) GSEA analysis highlighting Taz‐induced enriched gene sets of KEGG_UBIQUTIN_MEDIATED_PROREOLYSIS. D) Representative immunoblot analysis and quantification of the effect of Taz on PD‐L1 expression in the absence and presence of proteasome inhibitors MG‐132. Results are presented as mean ± SEM, two‐tailed unpaired *t*‐test. E) Representative immunoblot analysis of the effect of Taz on exogenous FLAG‐PD‐L1 expression F) Representative immunoblot analysis and G) quantification of exogenous FLAG‐PD‐L1 expression in HCT116 treated with Taz or DMSO control, 20 µg mL^−1^ cycloheximide (CHX) was incubated indicated time. Results are presented as mean ± SEM, two‐tailed unpaired *t*‐test. H) immunoblot analysis of ubiquitinated FLAG‐PD‐L1 after immunoprecipitation by FLAG‐protein beads in HCT116 cells transfected with indicated constructs. I) Immunoblot analysis of ubiquitinated FLAG‐PD‐L1 after immunoprecipitation by FLAG‐protein beads under in 293T and HCT116 cells transfected with indicated constructs. J,K) immunoblot analysis of Taz‐induced ubiquitinated FLAG‐PD‐L1 after immunoprecipitation by FLAG‐protein beads in I) 293T and J) HCT116 cells transfected with indicated constructs.40 ng mL^−1^ IFN‐γ was added in (A), (B), and (D) ns., not significant; *p > *0.05, ^*^ *p < *0.05, ^**^ *p < *0.01, ^***^ *p < *0.001.

To corroborate the influence of EZH2 inhibition on the stability of the PD‐L1 protein, we employed CHX to verify the change in the half‐life of PD‐L1 after Taz treatment. The half‐life of PD‐L1 proteins significantly extended after both chemical and genetic EZH2 inhibition, which provides additional evidence for the involvement of EZH2 in the regulation of PD‐L1 protein stability (**Figure** **2**A,B; Figure S2A–C, Supporting Information). Complementing these findings, RNA‐seq analysis elucidated substantial alterations in signaling pathways associated with protein stability regulation, particularly highlighting the UPS‐related pathways (Figure 2C), which are known to play key roles in the post‐translational modifications of the PD‐L1 protein. Consistently, the proteasome inhibitor MG‐132 and PSI were able to upregulate the expression of PD‐L1 in colorectal cancer, but not lysosome or autophagy inhibitor (Figure 2D; Figure S2D, Supporting Information), suggesting that EZH2 inhibition may primarily enhance PD‐L1 protein stability through its influence on the ubiquitin‐proteasome pathway, specifically in a proteasome‐dependent manner. In addition, we established FLAG‐PD‐L1 ectopic expression cell models in HCT116 cell lines (Figure S2E,F, Supporting Information), aiming to eliminate potential confounding factors related to transcriptional regulation. As anticipated, the administration of EZH2 inhibitors led to a significant upregulation of exogenous PD‐L1 expression (Figure 2E), supporting the existence of post‐transcriptional mechanisms through which EZH2 enhances PD‐L1 expression. Similarly, Taz enhanced the stability of exogenous PD‐L1 protein and extended its half‐life (Figure 2F,G). This collective evidence strongly indicates that EZH2 not only exerts its influence on PD‐L1 expression at the transcriptional level but also significantly impacts PD‐L1 protein stability, underscoring its multifaceted regulatory role in the modulation of PD‐L1 in cancer cells.

To further explore the regulatory role of Taz on the ubiquitin‐proteasome pathway of PD‐L1, we detected ubiquitinated PD‐L1 following Taz treatment. Taz treatment significantly reduced the ubiquitination of PD‐L1 (Figure 2H). Furthermore, previous reports have indicated that the ubiquitination of PD‐L1 primarily occurs at specific lysine residues.^[^ ^27^, ^28^ ^]^ Understanding the precise sites of ubiquitination could offer valuable insights into the mechanisms through which EZH2 regulates PD‐L1 protein stability. To investigate the specific effects of EZH2 inhibition on the ubiquitination modifications of PD‐L1, we generated ubiquitin mutants K48R and K63R (lysine to arginine) to target the ubiquitination sites. As expected, both the K48R and K63R mutants markedly reduced PD‐L1 ubiquitination, with a more pronounced reduction observed in the case of the K48R mutation (Figure 2I). These findings indicate that PD‐L1 is subject to concurrent regulation by ubiquitination modifications at both the K48 and K63 sites, with the K48 site playing a predominant role in this regulatory process. Upon treatment with the EZH2 inhibitor, we observed a significant decrease in the overall ubiquitination of PD‐L1 (Figure 2H,J,K), along with a notable reduction in K63‐mediated ubiquitination modifications (Figure 2J,K). Interestingly, the changes in ubiquitination at the K48 site were not statistically significant, suggesting that EZH2 inhibition primarily targets and attenuates K48‐mediated ubiquitination modifications. This selective impact on K48‐mediated ubiquitination is likely responsible for the observed enhancement of PD‐L1 protein stability.

### 2.3 USP22 is a Key Deubiquitinase (DUB) Involved in the Regulation of PD‐L1 Stability by EZH2

From the above results, it can be concluded that EZH2 inhibitors can regulate PD‐L1 expression through ubiquitination alteration. To explore the underlying mechanisms of how EZH2 regulates PD‐L1, we analyzed RNA‐seq data of HCT116 cells treated with 40 µм Taz for 48 h and identified 319 differentially expressed genes (DEGs) potentially involved in ubiquitination.^[^ ^29^ ^]^ Thirteen genes predicted to interact with PD‐L1^[^ ^30^ ^]^ were then identified as being differentially expressed after Taz treatment (Figure S3A, Table S1, Supporting Information). Three deubiquitinases, USP8, USP9X, and USP22, were upregulated after Taz treatment. To further identify which DUB regulates PD‐L1 stability, selected DUBs alone with OTUB1 and CSN5, which are known DUBs of PD‐L1,^[^ ^31^, ^32^ ^]^, were knocked down respectively and the effects of Taz on PD‐L1 expression under such conditions were detected. The results showed that only USP22 was correlated with PD‐L1 expression and was able to weaken Taz‐induced upregulation of PD‐L1 (Figure S3B, Supporting Information). USP22 is a member of the ubiquitin‐specific protease (USP) deubiquitinase family. It has been reported to deubiquitinate the intracellular domain of PD‐L1 with a C‐terminal fragment and suppress anticancer immunity in various cancers.^[^ ^33^, ^34^ ^]^ However, there is no research indicating whether EZH2 can regulate the stability of the PD‐L1 protein through its effect on USP22 expression.

**Figure 3:**
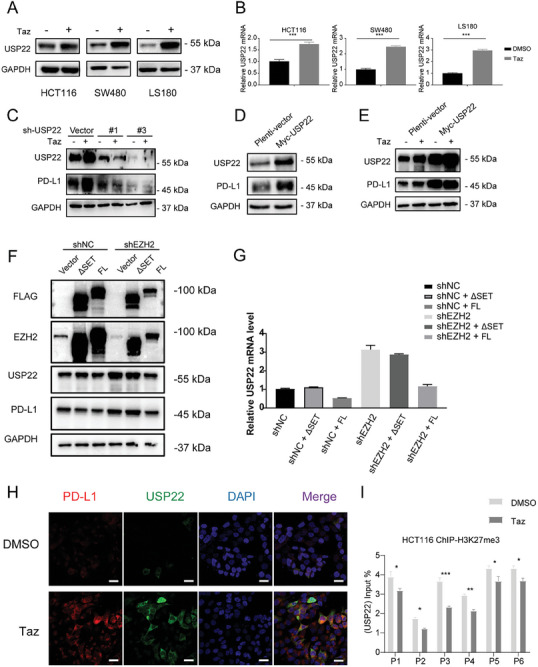
USP22 is a key deubiquitinase (DUB) involved in the regulation of PD‐L1 stability by EZH2. A) Representative immunoblot analysis and B) qPCR of USP22 in various colon cancer cell lines. Results are presented as mean ± SEM, two‐tailed unpaired *t*‐test. C) Immunoblot analysis of the USP22 and PD‐L1 protein expression in HCT116 cell after treatment with USP22 shRNAs or scrambled shRNA. D) Immunoblot analysis of the PD‐L1 protein expression after ectopic Myc‐USP22 overexpression in HCT116 cells. E) Representative immunoblot analysis of the PD‐L1 protein expression after ectopic Myc‐USP22 overexpression in HCT116 cells treated with Taz for 48 h and 40 ng mL^−1^ IFN‐γ for an additional 24 h. F) Immunoblot analysis of PD‐L1 and USP22 and G) qPCR analysis of USP22 mRNA in wild‐type or EZH2 knockdown cells following the exogenous transfection with empty, ΔSET, or FL EZH2 plasmids. H) Representative immunofluorescence staining of endogenous PD‐L1 and USP22 after Taz or DMSO treatment in HCT116 cells. Scale bar, 20 µm.I) H3K27me3 binding to the USP22 promoter was determined by chromatin immunoprecipitation‐qPCR in HCT116 cell lines. ΔSET: SET domain deletion. FL: full length. Results are presented as mean ± SEM, two‐tailed unpaired t‐test. ns., not significant; *p > *0.05, ^*^ *p < *0.05, ^**^ *p < *0.01, ^***^ *p < *0.001.

To validate this observation, we assessed the expression of USP22 following Taz treatment in various colon cancer cell lines. Taz enhanced USP22 expression at both the mRNA and protein levels in various colon cancer cell lines, including HCT116, SW480, LS180, and MC38 cells (**Figure** **3**A; Figure S3C,D, Supporting Information). USP22 knockdown led to decreased PD‐L1 expression and attenuated the upregulatory effect of EZH2 inhibition on PD‐L1 (Figure 3C; Figure S3E, Supporting Information). Conversely, ectopic expression of USP22 caused an enhancement in PD‐L1 protein expression, and further attenuating the upregulatory effect of Taz on PD‐L1 expression (Figure 3D,E). These findings suggest that EZH2 may regulate PD‐L1 through its enhancement of USP22 expression.

The SET domain (612–727aa) is the key catalytic region of EZH2 that mediates the methylation of histone proteins. To validate the relationship between the enzymatic activity of EZH2 and the expression of USP22 and PD‐L1, we transfected wild‐type and EZH2 knockdown HCT116 cell lines with either an empty vector, a SET domain deletion (ΔSET, 1–611aa), or a full‐length (FL) EZH2 overexpression plasmid. Overexpression of EZH2 could modestly inhibit the transcription of USP22 and compensate for the increased USP22 expression resulting from EZH2 knockdown (Figure 3F,G). In contrast, overexpression EZH2 lacking the SET domain did not significantly affect USP22 or PD‐L1 expression (Figure 3F,G). This suggests that EZH2 regulates USP22 expression through its SET domain‐mediated histone methylation activity.

The immunofluorescence results similarly indicated that Taz treatment upregulated the cellular expression of both USP22 and PD‐L1 (Figure 3H). The ChIP‐qPCR results demonstrated H3K27me3 modifications at the USP22 promoter region, and Taz treatment reduced H3K27me3 modifications in the promoter region, thereby promoting USP22 transcription (Figure 3I), which is consistent with the impact of the SET domain of EZH2 on USP22 expression. These findings indicate that EZH2 may control USP22 expression through its classical epigenetic mechanisms, thereby influencing PD‐L1 protein stability.

### 2.4 EZH2 Enhances the Protein Stability of PD‐L1 Through the Deubiquitination Effect Mediated by USP22

**Figure 4:**
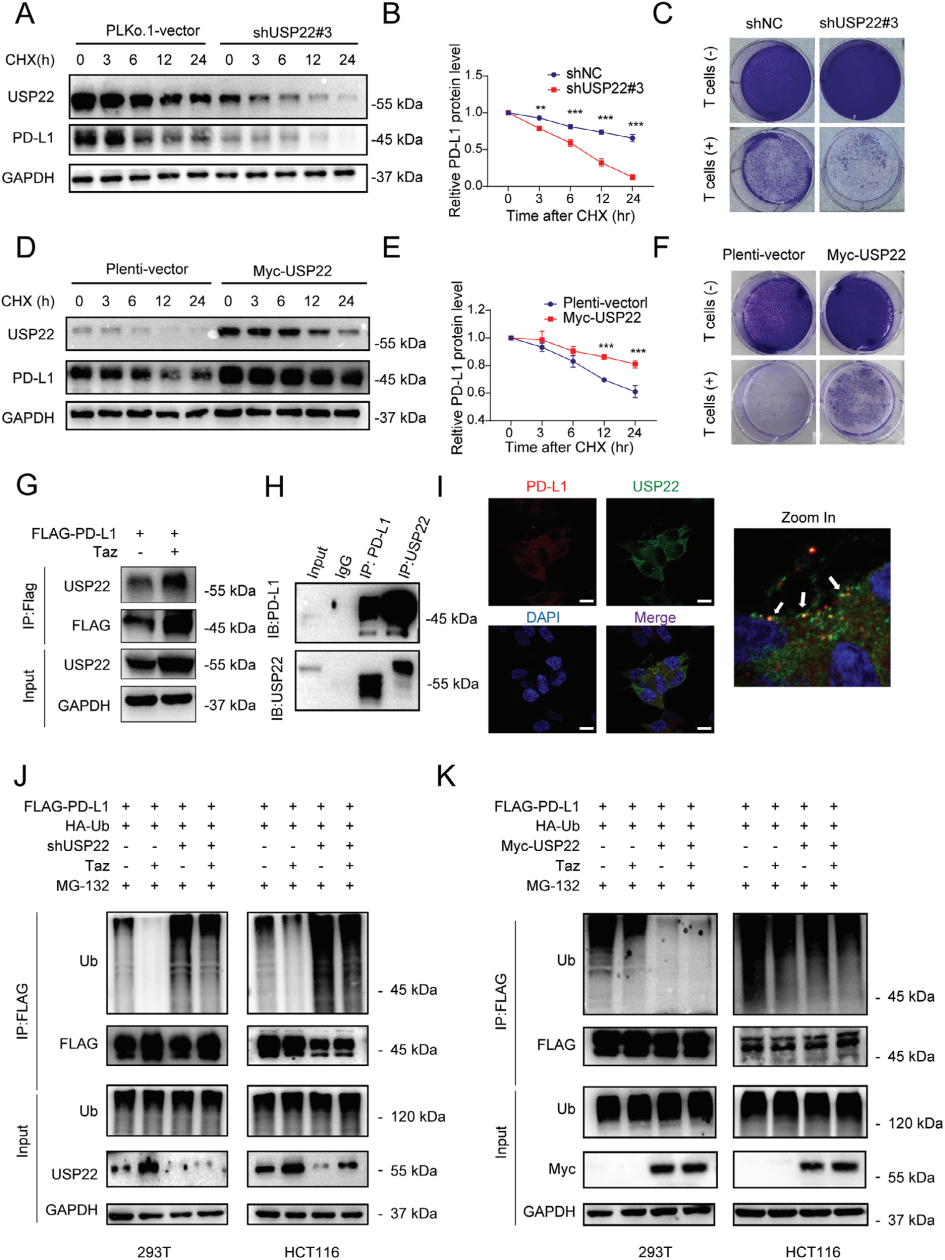
EZH2 enhances the protein stability of PD‐L1 through the deubiquitination effect mediated by USP22 A) Representative immunoblot and B) quantification analysis of endogenous PD‐L1 degradation in HCT116 treated with shUSP22 or scrambled shRNA, 20 µg mL^−1^ cycloheximide (CHX) was incubated indicated time. C) HCT116‐shUSP22 cells were cocultured with or without activated *T*‐cells at a ratio of 1:2 for 72 h and subjected to crystal violet staining. D) Representative immunoblot and E) quantification analysis of endogenous PD‐L1 degradation in HCT116 treated with ectopic Myc‐USP22 overexpression or vector plasmid, 20 µg mL^−1^ cycloheximide (CHX) was incubated indicated time. F) HCT116‐USP22 OE cells were cocultured with or without activated *T‐cells* at a ratio of 1:5 for 72 h and subjected to crystal violet staining. G) Representative immunoblot of immunoprecipitated FLAG‐PD‐L1 and the bound USP22. H) Representative immunoblot of Co‐IP analysis to confirm the interaction between USP22 and PD‐L1 I) Representative immunofluorescence staining of endogenous PD‐L1 and USP22 in HCT116 cells. Scale bar, 10 µm J,K) Immunoblot analysis of ubiquitinated FLAG‐PD‐L1 after immunoprecipitation by FLAG‐protein beads in J) 293T and K) HCT116 cells transfected with indicated constructs. ns., not significant; *p > *0.05, ^*^ *p < *0.05, ^**^ *p < *0.01, ^***^ *p < *0.001.

In HCT116 colon cancer cells, knocking down USP22 weakens the protein stability of PD‐L1 and resulted in a shorter half‐life, alone with attenuated resistance to *T*‐cell cytotoxicity (**Figure** **4**A–C; Figure S4A, Supporting Information). Conversely, ectopic USP22 expression enhanced the stability of PD‐L1 and prolonged its half‐life, as well as enhanced resistance to *T*‐cell cytotoxic effects and resulted in an increase in cell viability (Figure 4D–F; Figure S4B, Supporting Information).

As reported in previous literature, USP22 can regulate the stability of PD‐L1 through a direct interaction.^[^ ^34^ ^]^ Consistent with previous reports, USP22 can interact with both endogenous and exogenous PD‐L1 proteins (Figure 4G,H). In addition, according to the public dataset on CCLE, no significant correlation was found between USP22 and PD‐L1 transcription levels, but a significant positive correlation was observed at the protein level, which confirmed the role of USP22 in the post‐translational regulation of PD‐L1 stability (Figure S4C,D, Supporting Information). Additionally, immunostaining results revealed spatial consistency between PD‐L1 and USP22 in terms of their cellular expression (Figure 4I). We sought to determine whether USP22 catalyzes the deubiquitination of PD‐L1 and participates in regulating the deubiquitination effect of EZH2 inhibitors on PD‐L1. In both 293T and HCT116 cells, knocking down USP22 induced an increase in ubiquitination of PD‐L1 and further weakened the inhibitory effect of Taz on PD‐L1 ubiquitination (Figure 4J). Conversely, overexpressing USP22 reduced the ubiquitination modifications of PD‐L1, thereby diminishing the inhibitory effects of EZH2 inhibitors on PD‐L1 ubiquitination (Figure 4K). However, overexpression of ΔSET or FL (full‐length) EZH2 did not significantly affect PD‐L1 ubiquitination modification, which may be due to the enzymatic activity of EZH2 being redundant under normal conditions (Figure S4E, Supporting Information).

### 2.5 EZH2i Plays a Synergistic Anticancer Effect in Combination with Immunotherapy In vivo

**Figure 5:**
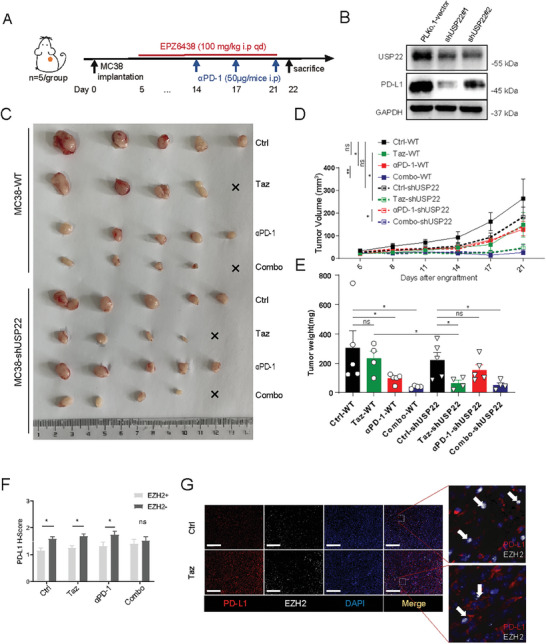
Taz enhances the sensitivity of tumors to immunotherapy in vivo. A) Working model of engraftment and treatment of the subcutaneous transplantation model of MC38. B) Representative immunoblot analysis of the effects of USP22 knockdown on PD‐L1 expression in MC38 cell after treatment with USP22 shRNAs or scrambled shRNA. C) Overview of tumors, D) tumor volume, and E) weights of tumor bulk. F) PD‐L1 H‐scores in EZH2^−^ or EZH2^+^ cells of subcutaneous MC38 transplant tumors in control or Taz groups. G) Representative immunofluorescence staining of EZH2 and PD‐L1 in MC38 transplant subcutaneous tumor of control or Taz groups. Scale bar, 200 µm. ns., not significant; *p > *0.05, ^*^ *p < *0.05, ^**^ *p < *0.01, ^***^ *p < *0.001.

EZH2 inhibitors upregulate PD‐L1 expression in tumor tissue, which may be one of the reasons limiting the efficacy of EZH2 inhibitors in solid tumors. To overcome this drawback of EZH2 inhibitors in tumor treatment, we applied immunotherapy combined with EZH2 inhibitors to test the feasibility of synergic treatment using a murine xenograft tumor model. Wild‐type MC38 or MC38 USP22‐knockdown subcutaneous tumor models were established in C57BL/6J mice. Taz and anti‐PD‐1 antibody were administered alone or in combination (**Figure** **5**A). Consistent with previous results, the knockdown of USP22 significantly reduced tumor PD‐L1 expression in a mouse model (Figure 5B). MC38‐ shUSP22#1 cell was used for the construction of subcutaneous model and the results demonstrated that the combination therapy, compared to Taz or α‐PD‐1 monotherapy, significantly suppressed tumor growth, showing a synergistic anticancer effect in vivo (Figure 5C–E). Notably, although knocking down USP22 only mildly reduced tumor growth, it significantly increased the sensitivity of tumors to Taz. Meanwhile, knocking down USP22 weakens the inhibitory effect of immunotherapy, and combination treatment did not yield further enhanced efficacy compared to Taz monotherapy. Additionally, we observed a significant negative correlation between EZH2 expression and PD‐L1 levels in the tumor microenvironment (TME). EZH2‐negative cell subpopulations exhibited higher PD‐L1 expression than EZH2‐positive cell subpopulations (Figure 5F,G).

**Figure 6:**
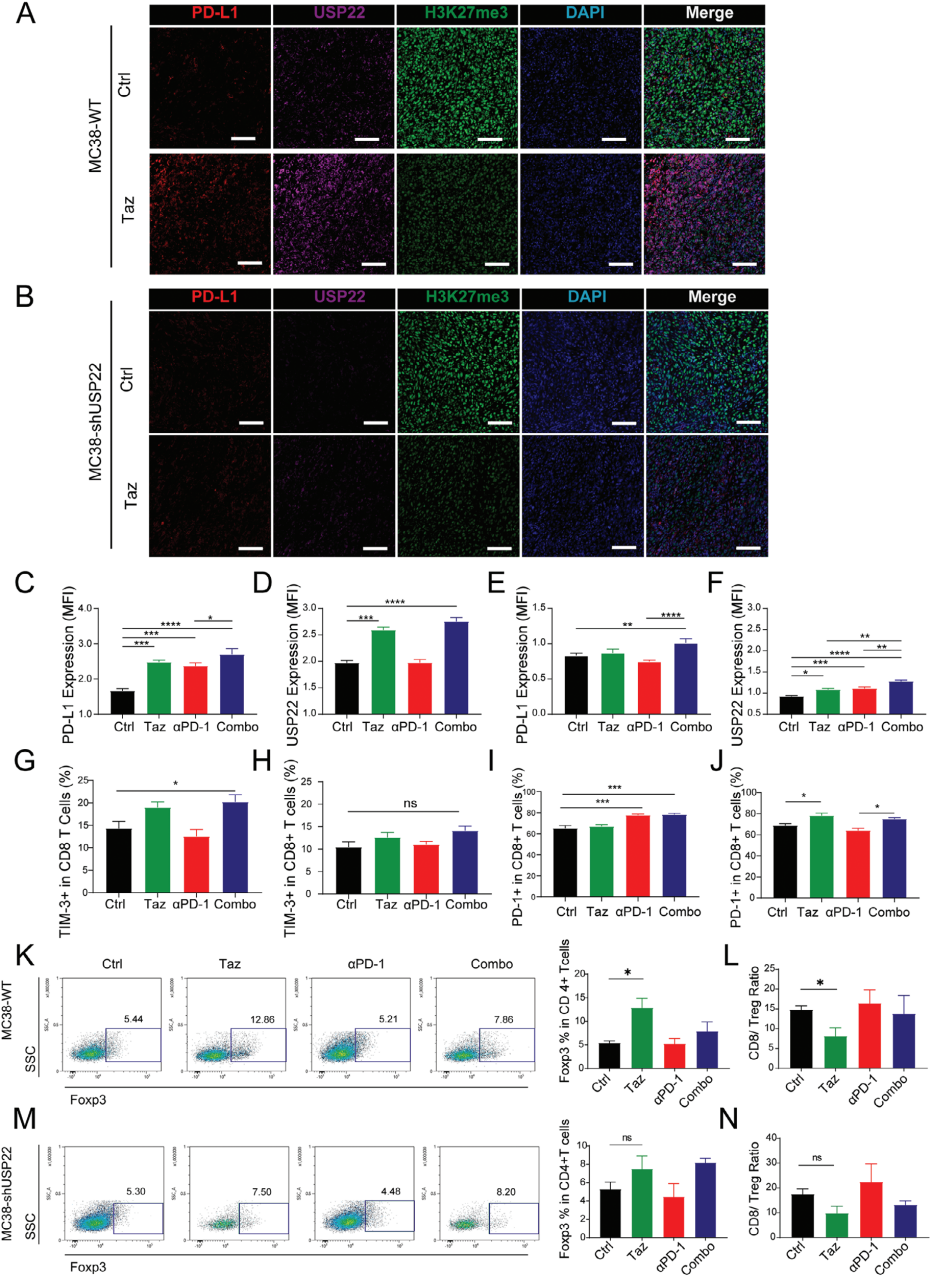
Inhibition of EZH2 and USP22 affects the tumor microenvironment. A) Representative immunofluorescence staining of H3K27me3, PD‐L1, and USP22 in MC38‐WT and B) MC38‐shUSP22 transplant subcutaneous tumor of control or Taz groups. Scale bar, 100 µm. C) Average fluorescence intensity of PD‐L1 and D) USP22 in MC38‐WT tumor microenvironment of different groups. E) Average fluorescence intensity of PD‐L1 and F) USP22 in MC38‐shUSP22 tumor microenvironment of different groups. G) The proportion of late‐stage exhausted TIM‐3^+^ cells among CD8^+^ *T*‐cells in MC38‐WT and H) MC38‐shUSP22 transplant subcutaneous tumor of different groups. I) The proportion of PD‐1^+^ cells among CD8^+^ *T*‐cells in MC38‐WT and J) MC38‐shUSP22 transplant subcutaneous tumor of different groups. K) The proportion of Foxp3^+^ Treg infiltration among CD4^+^ cells and L) CD8/Treg ratio in the draining lymph node of different groups. M) The proportion of Foxp3^+^ Treg infiltration among CD4^+^ cells and N) CD8/Treg ratio in the MC38‐shUSP22 draining lymph node of different groups. ns., not significant; *p > *0.05, ^*^ *p < *0.05, ^**^ *p < *0.01, ^***^ *p < *0.001.

We performed multiplex immunofluorescence to examine the microenvironment of MC38 transplant tumors (Figure S5, Supporting Information). Consistent with the results of the in vitro cell experiments, Taz treatment significantly upregulated PD‐L1 expression in the TME (**Figure** **6**A, C). Meanwhile, the expression of USP22 was also upregulated after Taz treatment (Figure 6A,D). Decreased PD‐L1 expression in the tumor environment after USP22 knockdown was found and the upregulation effects of PD‐L1 and USP22 induced by Taz or immunotherapy were also attenuated (Figure 6B,E,F), which suggested that USP22 is involved in EZH2‐mediated regulation of PD‐L1 expression.

Upon examination of exhausted T‐cells in the TME, treatment with Taz but not *α*‐PD‐1 immunotherapy increased the infiltration of TIM‐3+ late‐stage exhausted CD8^+^ *T*‐cells within the TME (Figure 6G). USP22 knockdown reduced the overall infiltration of late‐stage exhausted TIM‐3+ CD8^+^ *T*‐cells and attenuated the upregulatory effect induced by Taz or combination therapy (Figure 6H). However, an increase in early‐stage exhausted PD‐1 + CD8^+^ *T*‐cells predominantly occurred following anti‐PD‐1 immunotherapy but was less impacted by Taz‐mediated regulation (Figure 6I). After USP22 knockdown, overall infiltration of early‐stage exhausted PD‐1 + CD8^+^ *T*‐cell was comparable to the wild‐type MC38 tumor model, and t a mild tendency of enhancement of infiltration of PD‐1+ CD8^+^ *T*‐cell was observed after Taz treatment (Figure 6J).

Moreover, Taz treatment could upregulate Treg recruitment in tumor‐draining lymph nodes (tDLNs), reducing the CD8^+^/Treg ratio and thereby skewing the immune environment toward immunosuppression. Combination anti‐PD‐1 immunotherapy partially counteracted the pro‐immunosuppressive effect induced by Taz, restoring the CD8/Treg ratio (Figure 6K,L). However, after knocking down USP22, Taz no longer significantly influenced Treg recruitment or the CD8^+^/Treg ratio in the dDLNs (Figure 6M,N), which may be because knocking down USP22 weakened the immune‐suppressive effects mediated by PD‐L1. In addition, in the MC38‐WT subcutaneous model, Taz or PD‐1 treatment had little impact on CD8^+^infiltration or the expression of cytotoxic cytokines on CD8^+^ *T‐*cells, such as GZMB and perforin, However, in tumors with USP22 knockdown, there was no significant change in the overall expression of GZMB and perforin in CD8^+^ T‐cells after Taz treatment, except for an enhanced expression of perforin after αPD‐1 immunotherapy (Figure S6A–F, Supporting Information). Interestingly, we unexpectedly found that Taz treatment increased the infiltration of CD8^+^ *T*‐cells in tumors where USP22 had been knocked down but not in the combination therapy group (Figure S6F, Supporting Information). This phenomenon warrants further investigation. Additionally, we observed that Taz treatment resulted in an increasing trend of M1 macrophages, with no significant impact on M2 macrophages (Figure S6G,H, Supporting Information). In the USP22 knockdown model, although the overall proportion of M1 infiltration was not significantly different from the wild type, there were no significant differences in infiltration among the groups (Figure S6I,J, Supporting Information). Furthermore, the knockdown of USP22 reduced the infiltration of M2 macrophages, but no significant differences in M2 infiltration were observed with Taz treatment or immunotherapy. These phenomena may arise from the global effects of EZH2 inhibitors.

Based on these data, it comes out the following conclusions: The EZH2 inhibitor upregulates PD‐L1 expression in colon cancer cells, and this upregulatory effect is dependent on or at least partially reliant on enhanced PD‐L1 stability through USP22, primarily achieved through the regulation of PD‐L1 ubiquitination modifications. At the animal level, the EZH2 inhibitor can increase PD‐L1 levels in the tumor microenvironment and demonstrate a synergistic anticancer effect when combined with immune checkpoint blockade therapy.

### 2.6 The Predictive Value of USP22 and EZH2 for the Prognosis and Immune Infiltration of Colorectal Cancer

**Figure 7:**
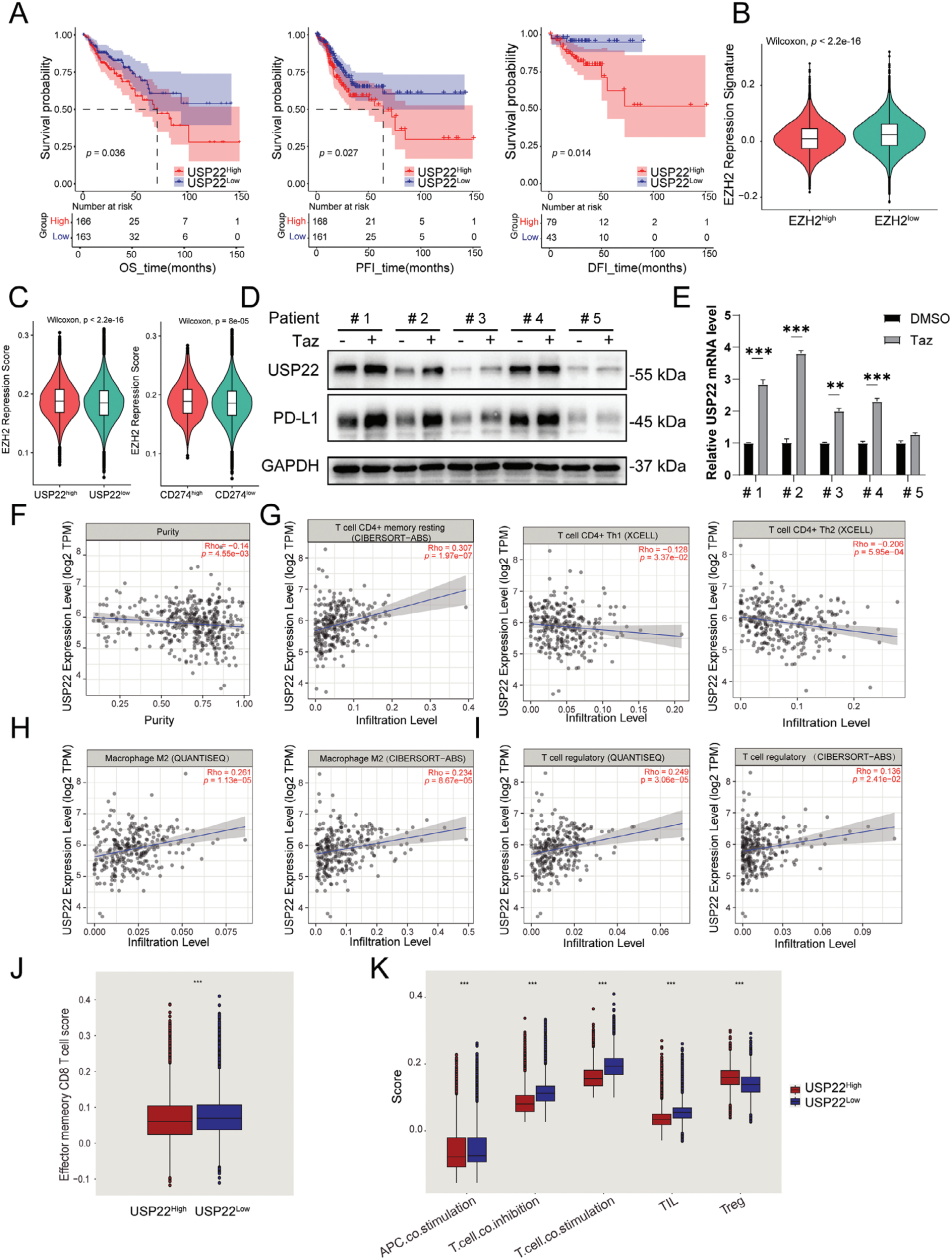
USP22 expression in clinical colorectal cancer samples. A) Survival analyses of USP22 in the TCGA‐COAD cohort by Kaplan–Meier estimator. The numbers below the figures denote the number of patients at risk in each group. OS: overall survival; DFI: disease‐free Free Interval; PFI: progression‐free interval, *p*‐value < 0.05. B) Violin plot representing EZH2 repression signature score significantly increased in EZH2^low^ colorectal cancer cells (Wilcoxon test, dataset: syn26844071), *p*‐value < 2.2×10^−16^. C) Violin plot representing EZH2 repression signature score significantly positively correlated with USP22 and CD274 expression in colorectal cancer cells (dataset: syn26844071), D) Representative immunoblot analysis and E) qPCR analysis of USP22 in primary cells derived from clinical samples of colorectal cancer. Primary cells were treated with Taz for 48 h and 40 ng/ml IFN‐γ for an additional 24 h. Results are presented as mean ± SEM, two‐tailed unpaired *t*‐test. F–I) Correlation of USP22 expression with immune infiltration level obtained from TIMER 2.0, including purity F), CD4^+^ *T*‐cells G), macrophages H), and T regulatory cells I) in TCGA‐COAD cohort. J,K) Immune infiltration scores significantly increased in USP22^low^ colorectal cancer cells (Wilcoxon test, dataset: syn26844071).

We evaluated the prognostic value of USP22 expression via Kaplan–Meier analysis in TCGA‐COAD (**Figure** **7**A). The prognosis analysis demonstrated that COAD patients with high USP22 expression had inferior overall survival (*p* = 0.036) in comparison to patients who had low USP22 expression. In addition, the increased USP22 expression exhibited a poor progression‐free interval (*p* = 0.027) and disease‐free interval (*p* = 0.014) in COAD patients. These results suggest that USP22 could serve as a potential biomarker for predicting patient treatment outcomes. To determine whether loss of EZH2 function was associated with USP22 expression in human COAD, a 75‐gene EZH2 repression signature was defined through intersecting EZH2 target genes (Table S2, Supporting Information) with differentially expressed genes (DEGs) upregulated after Taz treatment in HCT116 cells (Figure S7A and Table S3, Supporting Information). The EZH2 repression signature score was calculated in a human colorectal cancer scRNA‐seq dataset.^[^ ^26^ ^]^ We found that colorectal cancer cells with low expression of EZH2 had a higher EZH2 repression signature score (*p < *2.2×10^−16^, Wilcoxon) (Figure 7B). Simultaneously, the EZH2 repression signature score was positively correlated with the expression of USP22 (*p < *2.2×10^−16^, Wilcoxon) and PD‐L1 (*p < *8×10^−5^, Wilcoxon) (Figure 7C). Similar associations were also observed in two additional datasets (GSE166555 and GSE200997, Figure S7B,C, Supporting Information). These results indicated that in line with our experimental data, low EZH2 activity in COAD patients was also associated with increased expression of USP22 and PD‐L1. To validate the regulatory effects of EZH2 on USP22 and PD‐L1 in clinical specimens, primary tumor cells were isolated from colorectal cancer tissues and treated with Taz. The results indicate that EZH2 inhibition can upregulate USP22 and PD‐L1 expression in four of five primary cells (Figure 7D,E). Additionally, in a variety of solid tumors, including hepatocellular carcinoma, pancreatic cancer, gastric cancer, head and neck squamous cell carcinoma, and breast cancer, we observed a positive correlation between the EZH2 repression signature score and both PD‐L1 and USP22, suggesting that the regulatory effect of the EZH2‐USP22‐PD‐L1 axis may be widely present in solid tumors (Figure S7D, Supporting Information).

Then, we investigated the potential role of USP22 in immune cell infiltration in TCGA‐COAD using the TIMER 2.0 database (Figure 7F–I). In colorectal cancer, USP22 expression was negatively correlated with tumor purity, which was an indicator of prognosis,^[^ ^35^ ^]^ as determined by analyzing the ratio of stromal and infiltrated immune cells (*r = *−0.14, *p = *4.55×10^−3^, Spearman) (Figure 7F). USP22 expression was positively correlated with CD4^+^ T memory resting cells (*r = *0.307, *p = *1.97×10^−7^, Spearman) and negatively correlated with both Th1 (*r* = −0.128, *p* = 3.37×10^−2^, Spearman) and Th2 (*r* = −0.206, *p* = 5.95×10^−4^, Spearman) helper cells (Figure 7G). Furthermore, USP22 expression was positively correlated with immune‐suppressive M2 macrophages (*r* = 0.261, *p* = 1.13×10^−5^ for the QUANTISEQ algorithm; *r* = 0.234, *p* = 8.67×10^−5^ for the CIBERSORT‐ABS algorithm, Spearman) (Figure 7H) as well as Treg infiltration (*r* = 0.249, *p* = 3.06×10^−5^ for the QUANTISEQ algorithm; *r* = 0.136, *p* = 2.41×10^−2^ for the CIBERSORT‐ABS algorithm, Spearman) (Figure 7I). We also evaluated the immune infiltration scores in a human colorectal cancer scRNA‐seq dataset^[^ ^26^ ^]^ through gene set variation analysis (GSVA) with two immune gene lists.^[^ ^36^, ^37^ ^]^ The analysis showed that high USP22 expression was related to lower memory CD8^+^ *T*‐cell scores (^***^ *p* < 0.001, Wilcoxon) (Figure 7J), and high USP22 expression was linked to an increase in *T*‐cell co‐inhibitory molecule expression, a decrease in TIL infiltration, and a higher Treg ratio (^***^ *p* < 0.001, Wilcoxon) (Figure 7K).

Transcriptomic analysis reveals that Taz activates a range of molecules involved in antigen presentation, including human leukocyte antigen (HLA) molecules and transporter associated with antigen processing (TAP), suggesting that EZH2 inhibition may also enhance antigen presentation‐related processes, thereby boosting tumor immunogenicity (Figure S8A, Supporting Information). Additionally, low EZH2 expression was correlated with elevated immune checkpoint expression and diminished MHC‐I molecule expression (^**^ *p* < 0.05, ^***^ *p* < 0.001, Wilcoxon) (Figure S8B, Supporting Information). Lower EZH2 expression was associated with diminished infiltration of activated and memory CD8^+^ *T‐cells*, a reduction in CD4^+^ *T*‐cell infiltration especially within the Th2 cell subtype, and an increase in Treg cell infiltration (^***^ *p* < 0.001, Wilcoxon) (Figure S8C, Supporting Information). This composite evidence suggests that low EZH2 expression and high USP22 expression may pertain to immune suppression, aligning with our research observations. Of note, certain immune activation‐associated molecules, e.g., APC co‐stimulatory molecules and *T*‐cell co‐stimulatory molecules, are also upregulated when USP22 expression is high. This seems to indicate that the influence on the immune microenvironment of USP22 extends beyond mere inhibitory effects, hinting at more intricate regulatory mechanisms that warrant further investigation.

## 3 Discussion

This study further identifies additional regulatory effects of EZH2 on PD‐L1, involving post‐translational modifications and protein stability mediated by USP22. Based on this finding, the clinical relevance of EZH2, PD‐L1, and USP22 is further clarified, and a combinational strategy is proposed for clinical practice.

Epigenetics is a crucial regulatory mechanism by which cells dynamically adapt to changes in their microenvironment. The histone methyltransferase EZH2 is widely recognized as a tumor‐promoting regulatory molecule.^[^ ^38^ ^]^ Despite the abnormal expression of EZH2 in tumors, EZH2 inhibitors are only effective in limited types of tumors, and clinical trials in most solid tumors appear unsatisfactory. The reason may be that EZH2 inhibitors also dynamically affect the tumor microenvironment, such as reprogramming the metabolic profile and/or weakening antitumor functions, such as producing an immunosuppressive effect by enhancing PD‐L1 expression.^[^ ^15^, ^16^, ^39^ ^]^ Therefore, this study indicates that combination with immune therapy can compensate for the immunosuppressive effect of EZH2 inhibitors and exert synergistic antitumor effects. In addition, EZH2 is positively correlated with MSI‐H or TMB in pan‐cancer,^[^ ^40^, ^41^, ^42^ ^]^ indicating a greater therapeutic potential for EZH2 inhibitors as well as higher sensitivity to immunotherapy, thereby further implying the rationality of the combination approach. In addition, recent studies have reported that PD‐L2 expressed by dendritic cells (DCs) can also modulate tumor immunity and attenuate the sensitivity of tumors to immunotherapy.^[^ ^43^ ^]^ Our transcriptome data found that Taz can also upregulate PD‐L2 on colon tumor cells; however, despite this upregulation at the transcript level, PD‐L2 expression remains low at the protein level. Therefore, further exploration is needed to determine whether EZH2 can similarly affect tumor immunity through PD‐L2 modulation (data not shown).

It was wildly reported targeting EZH2 can affect systemic immunity, beyond tumor cells.^[^ ^44^ ^]^ Our study discovered that intervening with EZH2 or USP22 can affect the infiltration of exhausted CD8+ effector *T‐cells* in the tumor microenvironment, particularly those expressing late‐stage exhausted TIM3+ CD8^+^ *T*‐cells. Considering that PD‐1 expression is upregulated immediately following *T*‐cell activation, TIM‐3+ *T*‐cells are more indicative of immunosuppressive effects.^[^ ^45^ ^]^ Therefore, Inhibition of EZH2 can induce immunosuppressive effects at the level of immune cells. Moreover, given that USP22 is positively correlated with M2 macrophage infiltration in public datasets, our study indeed found that knockdown of USP22 can reduce M2‐type macrophage infiltration. However, in this study, Taz treatment unexpectedly resulted in decreased M2 macrophage infiltration and increased M1 infiltration. Building upon our previous research, EZH2 inhibition can promote macrophage polarization toward the M1 phenotype through the transcription factor STAT3^[^ ^46^ ^]^. Therefore, in the condition of in vivo experiments, the immunosuppressive effects mediated by the upregulation of USP22 and PD‐L1 following Taz treatment may be overshadowed by the direct impact of EZH2 on macrophage polarization, leading to the observed phenomenon of increased M1 macrophage differentiation following Taz treatment, which is inconsistent with the public data results. This also suggests that, although this study primarily focuses on the enhanced effects of EZH2 inhibition on tumor cell immune checkpoints, targeting EZH2 in vivo can lead to more complex and dynamic effects, which still require further exploration in the future.

The proportion and abundance of PD‐L1 expression in tumor tissue may partially determine the benefits of immune checkpoint therapy.^[^ ^47^ ^]^ In hepatoma, anti‐PD‐1 immunotherapy can abolish the tumor‐promoting effect induced by upregulated PD‐L1 expression in vivo. However, enhanced PD‐L1 expression alone does not confer additional therapeutic benefit from immunotherapy.^[^ ^48^ ^]^ Thus, antitumor strategies that enhance PD‐L1 expression should be preferentially selected in combination with immunotherapy. Meanwhile, PD‐L1 expression is closely regulated at the protein level, including but not limited to glycosylation, phosphorylation, and ubiquitination. Multiple deubiquitinases, such as COP9 signalosome subunit 5 (CSN5), OTUB1, USP2, USP7, and USP22, have been reported to stabilize the PD‐L1 protein and augment its expression.^[^ ^31^, ^33^, ^49^, ^50^, ^51^ ^]^ Among them, USP22 has garnered increasing attention as a highlighted molecule that extensively participates in regulating various processes of tumor development and progression by interacting with and stabilizing key components such as HIF‐1α, HSPA5, the Wnt/β‐catenin pathway, and XPC.^[^ ^52^, ^53^, ^54^ ^]^ On the other hand, in colorectal cancer, loss of USP22 can activate the mTOR/PI3K/Akt pathway, exacerbating the tumor progression caused by Apc mutations in colorectal cancer.^[^ ^55^ ^]^ This contributes to the complex role of USP22 in tumors, displaying both pro‐tumorigenic and anti‐tumorigenic effects. In addition, USP22 was also reported to regulate tumor immunity and impact the antitumor immune response.^[^ ^56^ ^]^ USP22 physically interacts with the C‐terminus of the PD‐L1 protein and CSN5, a known DUB of PD‐L1, therefore deubiquitinates and stabilizes PD‐L1.^[^ ^33^, ^34^ ^]^ In addition, USP22 can also deubiquitinate and stabilize the SPI1 protein, thereby enhancing PD‐L1 transcription and expression.^[^ ^47^ ^]^ In addition, USP22 regulates other targets impacting tumor immunity. For instance, USP22 stabilizes Foxp3 and CD73, enhancing iTreg expression and affecting extracellular adenosine, respectively, thus suppressing tumor immunity.^[^ ^57^, ^58^, ^59^ ^]^ Concurrently, in prostate cancer, the absence of USP22 upregulates the infiltration of myeloid cells and promotes the infiltration of *T‐cells* and NK cells, improving the response to immune therapy and inhibiting the metastasis of pancreatic cancer cells in a *T*‐cell‐dependent manner.^[^ ^60^ ^]^ So far, among all the identified DUBs toward PD‐L1, how the cells choose the given DUB still needs to be determined.

As key members of the ubiquitin–proteasome system, DUBs play key roles in numerous cellular processes that are highly relevant to oncology, autoimmune disorders, and neurodegeneration.^[^ ^61^ ^]^ The clinical development of DUB inhibitors is challenging, and most DUB inhibitors are in preclinical development.^[^ ^61^, ^62^, ^63^, ^64^ ^]^ Two DUB small molecule inhibitors (VLX1570 and KSQ‐4279) have entered phase I clinical trials in malignant tumors (NCT02372240 and NCT05240898). However, these clinical trials have not yet achieved sufficiently good results.^[^ ^65^ ^]^ suggesting a long path ahead in developing DUB inhibitors for cancer treatment.

Indeed, this study has several limitations. First, due to the complexity of the regulatory mechanisms governing PD‐L1 expression, EZH2, and USP22 might not fully explain the differential expression of PD‐L1. Second, although we observed moderate enrichment of H3K27me3 at the USP22 promoter region, EZH2 may exert additional yet unelucidated regulatory mechanisms on USP22. Third, it will be necessary to establish a USP22 knockout model to further focus on the EZH2‐USP22‐PD‐L1 regulatory axis. Finally, comprehensive clinical cohort data are further needed to fully determine the predictive role of the EZH2‐USP22‐PD‐L1 regulatory axis in immunotherapy or EZH2 inhibition.

In summary, our study revealed a novel regulatory effect of EZH2 on the stability of the PD‐L1 protein, uncovered the resistance mechanism of the EZH2‐USP22‐PD‐L1 axis to EZH2 inhibitor treatment, and validated the synergistic anticancer capability of EZH2 inhibitors with immunotherapy. USP22 expression may be a potentially predictive indicator of the anticancer effects of EZH2 inhibitors. These findings could aid in the precise selection of patients in clinical settings, enabling personalized treatment strategies.

## 4 Experimental Section

### Cell Culture

HCT116 cells were cultured in IMDM (Gibco), LS‐180 cells were cultured in MEM (Gibco), SW480 cells were cultured in RPMI‐1640 medium (Gibco), and MC38 cells were cultured in DMEM (Gibco) supplemented with 10% fetal bovine serum (FBS, GIBCO), 50 IU mL^−1^ penicillin, and 50 mg mL^−1^ streptomycin (GIBCO). All cell lines were purchased from the Cell Resources Center of Peking Union Medical College (Beijing, China) and were cultured at 37 °C in a humidified incubator in the presence of 5% CO_2_.

### Reagents and Antibodies

EPZ‐6438 (S7128) and GSK‐126 (S7061) were purchased from Selleckchem. Anti‐PD‐1 (clone RMP1‐14, BE0146) antibody was purchased from Bioxcell. The primary antibodies used in immunohistochemistry, western blot and confocal assays included antibodies against PD‐L1 (#13684, Cell Signaling Technology; # 64988 Cell Signaling Technology; ab213524, Abcam), EZH2 (#5246, Cell Signaling Technology), Tri‐Methyl‐Histone H3 (Lys27) (#9733, Cell Signaling Technology), CD8 (#98941, Cell Signaling Technology), USP22 (ab195289, Abcam), GZMB (ab289888, Abcam), and perforin (ab16074, Abcam). MG132 (HY‐13259) and CHX (HY‐12320) were purchased from MCE (Shanghai).

### RNA Interference

shRNA plasmids were constructed using the pLKO.1 vector plasmid, and the target sequence for shRNA was derived from Thermo Fisher Scientific. The USP22, EZH2 ΔSET, and EZH2 full‐length overexpression plasmid was purchased from GENECHEM (Shanghai, China). To obtain stable cell lines, the lentiviral expression vector and packaging vectors (pMD2. G and psPAX) were transfected into HEK293T‐cells to produce the virus. After collecting the viral supernatant fractions at 48 and 72 h, target cells were infected and selected with puromycin.

### Real‐Time Quantitative PCR (Real‐Time qPCR)

Real‐time qPCR was performed using the Applied Biosystems QuantStudio 5 system (Applied Biosystems, CA, USA). Actin beta (ACTB) was included as a housekeeping gene control to normalize expression levels. The primers utilized for qPCR are as follows:

**Table:** 

Species	Target	Primer sequence (5′–3′)
Human	ACTB	F: TGTTACAGGAAGTCCCTTGCC
		R: ATGCTATCACCTCCCCTGTGTG
	USP22	F: GGAAAATGCAAGGCGTTGGAG
		R: GGAAAATGCAAGGCGTTGGAG
	USP7	F: CCCTCCGTGTTTTGTGCGA
		R: AGACTAAGGTGCAGTACCAGA
	USP9X	F: TTGCCTTGATTCCAACAGCC
		R: AGTCGCCTGAGTGTTTAGCT
	CSN5	F: CACTGAAACCCGAGTAAATGC
		R: ACATCAATCCCAGAAAGCCAG
	CD274	F: TCACTTGGTAATTCTGGGAGC
		R: CTTTGAGTTTGTATCTTGGATGCC
Mouse	ACTB	F:AGGGTGTGATGGTGGGAATG
		R: CCAGTTGGTAACAATGCCATGT
	CD274	F:GCTCCAAAGGACTTGTACGTG
		R:TGATCTGAAGGGCAGCATTTC
	USP22	F:CTCCCCACACATTCCATACAAG
		R:TGGAGCCCACCCGTAAAGA

### Chip‐qPCR

HCT116 cells plated in 10 cm dishes were treated with DMSO or Taz for 48 h. Then, the cells were fixed with 1% formaldehyde for 10 min at room temperature. To stop the reaction, glycine was added to a final concentration of 0.125 m at room temperature for 5 min. Cells were scraped into cold PBS with a proteinase inhibitor, and the contents of each group were transferred to a 15 mL tube. A ChIP assay was performed using a Simple ChIP Plus Enzymatic Chromatin IP Kit (Magnetic Beads) (Cat#: 9005, Cell Signaling Technology) and anti‐histone H3K27me3 (Cat#: 9733, Cell Signaling Technology) according to the procedures provided by the manufacturer. The final ChIP DNA samples were then used as templates in qPCRs. Primers were designed based on the different promoter regions shown in Figure S5C (Supporting Information).

### Western Blot

Western blotting was performed using the cell lysate according to standard protocols. Briefly, proteins were separated by SDS‐PAGE, transferred to nitrocellulose filter membranes, and subjected to immunoblotting with antibodies. All antibodies were validated by the commercial vendor. Densitometric analyses of protein bands were performed using ImageJ software.

### Immunofluorescence and Confocal Microscopy

HCT116 cells were fixed in 4% paraformaldehyde for 15 min and then kept in a blocking buffer for 1 h. Then, the cells were incubated with primary antibodies overnight at 4 °C, followed by staining with secondary antibodies. Nuclei were stained with DAPI. After that, the cells were visualized using an LSM900 laser scanning confocal microscope.

### Flow Cytometry

Cells were stained with LIVE/DEAD Violet (Life Technologies) before antibody staining. The following fluorophore‐conjugated antibodies were used (BioLegend): CD45, CD3, CD4, CD8, Foxp3, CD274, and CD279.

For flow cytometric analyses of TILs, excised tumors were minced finely with a scalpel blade in a Petri dish, and single‐cell suspensions were obtained following protocols of Tumor Dissociation Kit, mouse (130‐096‐730, Miltenyi Biotec) and perform antibody staining.

### *T* Cell‐Mediated Tumor Cell Killing assays

*T*‐cell‐mediated tumor cell killing assays were performed as previously described.^[^ ^66^ ^]^ Primary human *T‐cells* isolated from healthy human peripheral blood were cultured in ImmunoCult‐XF *T*‐cell expansion medium (10981; STEMCELL Technologies) and activated with ImmunoCult Human CD3/CD28/CD2 *T*‐cell activator (10970; STEMCELL Technologies) and IL‐2 (10 ng mL^−1^; PeproTech, Rocky Hill, NJ, USA) were added to the culture medium and maintained for one week following the manufacturer's protocols. Activated *T‐cells* were supplied with anti‐CD3 antibody (100 ng mL^−1^; 16–0037; Thermo Fisher Scientific) and IL‐2 (10 ng mL^−1^) cocultured with HCT116 stable cells at a 2:1 to 5:1 ratio for 72 h. After that, the cells were washed with PBS three times to remove *T‐cells*, followed by staining with crystal violet. Finally, the dried plates were scanned and quantified.

### RNA Sequencing

RNA‐seq was carried out in HCT116 cells treated with DMSO or Taz. Briefly, total RNA was extracted with TRIzol reagent, and the amounts and integrity of RNA were assessed using the RNA Nano 6000 Assay Kit of the Bioanalyzer 2100 system (Agilent Technologies, CA, USA). cDNA libraries were prepared and sequenced by the Illumina NovaSeq 6000. After quality control, the clean reads were mapped to the hg38 human reference genome using HISAT2 (v2.0.5). FeatureCounts (v1.5.0‐p3) were used to count the read numbers mapped to each gene. Differential expression analysis was performed using the DESeq2 R package (1.20.0). The resulting *p* values were adjusted using Benjamini and Hochberg's approach for controlling the false discovery rate. *p* adj  < 0.05 was set as the threshold for significantly differential expression. Gene enrichment analysis was conducted using gene set enrichment analysis (GSEA) software (4.2.3) with the Hallmark gene set (MSigDB) (http://www.broadinstitute.org/gsea/index.jsp). The accession number for the RNA‐seq data reported in this paper is NCBI GEO: GSE243955.

### Patient Tumors and Prime Cancer Cell Establishment

Colon cancer samples were obtained from the general surgery department of Peking University Third Hospital with protocols approved by the local ethical committee. Tumor tissue was extensively washed with PBS and finely minced, then transferred into a 25 cm^2^ culture flask at 37 °C, with 5% CO_2_. The culture medium used RPIM 1640 medium supplemented with 15% FBS, and 2% Pen–Strep solution, and 0.2% mycoplasma. After 5–10 days, controlled trypsinizations were done to preferentially remove the contaminating fibroblasts. The cells were routinely passed once a week, and the medium was changed twice in between.

### Data Analysis

Survival analyses (overall survival (OS), disease‐free interval (DFI), and progression‐free interval (PFI)) in the TCGA‐COAD cohort were conducted between high and low USP222 expression groups (by median expression level) through Kaplan–Meier analysis with the log‐rank test using R packages survival and survminer. The surv_cutpoint function was used to determine the optimal cutoff value. The log‐rank test was used to calculate the significance of survival time differences (*p*‐value <  0.05).

The correlation of USP22 with immune infiltration levels, including CD4^+^ T‐cells, macrophages, and T regulatory cells, in COAD was analyzed with the Tumor Immune Estimation Resource (TIMER 2.0, http://timer.cistrome.org/).^[^ ^67^ ^]^ The “Purity Adjustment” option, which uses the partial Spearman's correlation, was selected to perform this association analysis.

scRNA‐seq data were downloaded through Synapse under the accession codes syn26844071 (https://www.synapse.org/#!Synapse:syn26844071/)^[^ ^26^ ^]^ and and GEO database^[^ ^68^, ^69^, ^70^, ^71^, ^72^, ^73^, ^74^ ^]^. For each dataset, tumor epithelial cells were selected for subsequent analysis. The scRNA‐seq data analysis was performed using Seurat (version 4.1.1) to conduct a standard process with R software (version 4.2.1). The data were normalized (NormalizeData) and scaled (ScaleData). Harmony software (version 0.1.0) was used to correct the batch effect. According to the expression of EZH2 or USP22, the cells were divided into high and low‐expression groups by median expression level. In Figure S1C (Supporting Information), the AddModuleScore function in the Seurat package with default parameters was used to obtain six immune‐related signaling pathway scores with the hallmark gene set (MSigDB). The target genes of EZH2 in Figure S7A (Supporting Information) were downloaded from the hTFtarget database.^[^ ^75^ ^]^ The EZH2 repression signature score was calculated with the AUCell package. The gene set variation analysis (GSVA) package (version 1.44.2) was used for single‐sample gene set enrichment analysis (ssGSEA) analysis to obtain immune scores using the gene list in TISIDB^[^ ^36^ ^]^ (Figure 7J; Figure S8C, Supporting Information) and reported immune signatures (Figure 7K; Figure S8B, Supporting Information),^[^ ^37^ ^]^ respectively.

### Multiplex IHC Staining Protocol

Paraffin sections of formalin‐fixed MC38 subcutaneous tumor xenograft tumors. An Opal 7‐color kit (Akoya Bioscience, NEL811001KT) was used for multiplex IHC. In the first round, antigen was retrieved with a pressure cooker (EDTA pH 9.0) at 125 °C for 3 min. Slides were cooled to room temperature (RT), washed with TBST with 0.5% Tween (3 times, 3 min), and incubated with H_2_O_2_ (3%) for 10 min. Slides were washed and blocked with blocking buffer (VECTASHIELD, H‐1400) for 10 min. CD8 (#98941, CST) was incubated at RT for 40 min. Slides were washed, and an HRP‐conjugated secondary antibody was incubated at RT for 10 min. TSA dye (1:100) was applied for 10 min after washes. This was repeated five more times using the following antibodies: PD‐L1 (# 64988 Cell Signaling Technology), Tri‐Methyl‐Histone H3 (Lys27) (#9733, Cell Signaling Technology), CD8 (#98941, Cell Signaling Technology), USP22 (ab195289, Abcam), GZMB (ab289888, Abcam), perforin (ab16074, Abcam), CD86 (ER1906‐01, HUABIO), CD206 (#24595, Cell Signaling Technology), F4/80 (#70076, Cell Signaling Technology), PD‐1(#84651, Cell Signaling Technology), TIM‐3 (#83882, Cell Signaling Technology). For the second and subsequent rounds, antigen retrieval was performed in EDTA (pH 6.0) buffer using a microwave (100–150 mW, 15 min). Nuclei were stained with DAPI (PerkinElmer) and mounted with medium (Solarbio, C0065). Anti‐rabbit (PV6001) or anti‐mouse (PV6000) secondary antibodies (Zhongshan Jinqiao Biotechnology) were used at a 1:1000 dilution.

### Multiplex IHC Imaging and inForm Analysis

The interface of the tumor and stroma was identified by pathological features. Slides were imaged using a Vectra Polaris automated multispectral microscope (Akoya Bioscience). Whole slide scans were performed using the ×10 objective lens. Acquired images were analyzed with inForm tissue finder software (Akoya bioscience) for tissue‐component segmentation and chemotactic score determination. CD8, PD‐1, TIM‐3, F4/80, CD86, and CD206 were quantified by the percentage of positive cells. The expression of PD‐L1, USP22, H3K27me3, GZMB, and perforin was quantified by mean fluorescence intensity (MFI).

### Mice

C57BL/6J mice (6–8 weeks old, body weight 18–20 g) were purchased from Beijing Vital River Laboratory Animal Technology Co., Ltd, China. All mice were maintained in specific‐pathogen‐free conditions with approval by the Institutional Committee of Peking Third Hospital. The food of all animals was in accordance with the standard diet for rodents. The animals used in the study were compliant with all relevant ethical regulations regarding animal research.

### Tumor Models and Treatments

In the subcutaneous tumor model, 6‐ to 8‐week‐old C57BL/6J mice were injected with 1 × 10^6^ MC38 colon carcinoma cells. When tumors grew to measurable levels, mice were randomly assigned and intraperitoneally injected with vector control, EPZ‐6438 (100 mg kg^−1^), anti‐PD‐1 antibody (50 µg pre‐mouse) and combination treatment. Tumor volumes (mm^3^) were calculated and recorded using the formula (longest diameter) × (shortest diameter)^2^ × 0.5.

### Quantification and Statistical Analysis

For the analysis of tumor growth data, an overall difference at each data collection point was evaluated using one‐way ANOVA. The fairness of mouse randomization was ensured by checking the *p‐*value of data collected on the initial day, with no experiments showing a clear bias toward any specific group regarding starting tumor volume. The assumptions of ANOVA testing were verified to ensure that the model assumption was not severely violated. All between‐group comparisons employed one‐way ANOVA and Dunnett's multiple comparison test or two‐tailed *t*‐test to ascertain significant differences. A *p‐*value < 0.05 was regarded as statistically significant.

All analyses and data visualization were conducted using R (http://www.r‐project.org, version 3.5.1) or GraphPad Prism software (GraphPad Software, Inc., version 8.0).

### Ethics Approval Statement

All sampling and experimental steps in this study were approved by the Ethics Committee of Peking University Third Hospital (License No. M2021013 and No. S2022234). Informed consent was obtained from all individuals included in this study.

## Conflict of Interest

The authors declare no conflict of interest.

## Supporting information

Supporting Information

Supporting Information

## Data Availability

The data that support the findings of this study are available on request from the corresponding author. The data are not publicly available due to privacy or ethical restrictions.
