# Supplementary material for: EZH2 Inhibition Enhances PD‐L1 Protein Stability Through USP22‐Mediated Deubiquitination in Colorectal Cancer
Source: Adv Sci (Weinh). 2024 Mar 22;11(23):2308045. doi: 10.1002/advs.202308045 (PMC11187912; doi:10.1002/advs.202308045)
Supplement: Supplementary file 1 — Supporting Information [file ADVS-11-2308045-s002.pdf]

ADVANCED SCIENCE
Open Access

Supporting Information

for *Adv. Sci.*, DOI 10.1002/advs.202308045

EZH2 Inhibition Enhances PD-L1 Protein Stability Through USP22-Mediated Deubiquitination in Colorectal Cancer

Jiaqi Huang, Qianqian Yin, Yuqing Wang, Xin Zhou, Yunyun Guo, Yuanjun Tang, Rui Cheng, Xiaotong Yu, Jie Zhang, Chen Huang, Zhanya Huang, Jianlin Zhang, Zhengyang Guo, Xiao Huo, Yan Sun, Yanfang Li, Hao Wang, Jianling Yang* and Lixiang Xue**

Figure S1

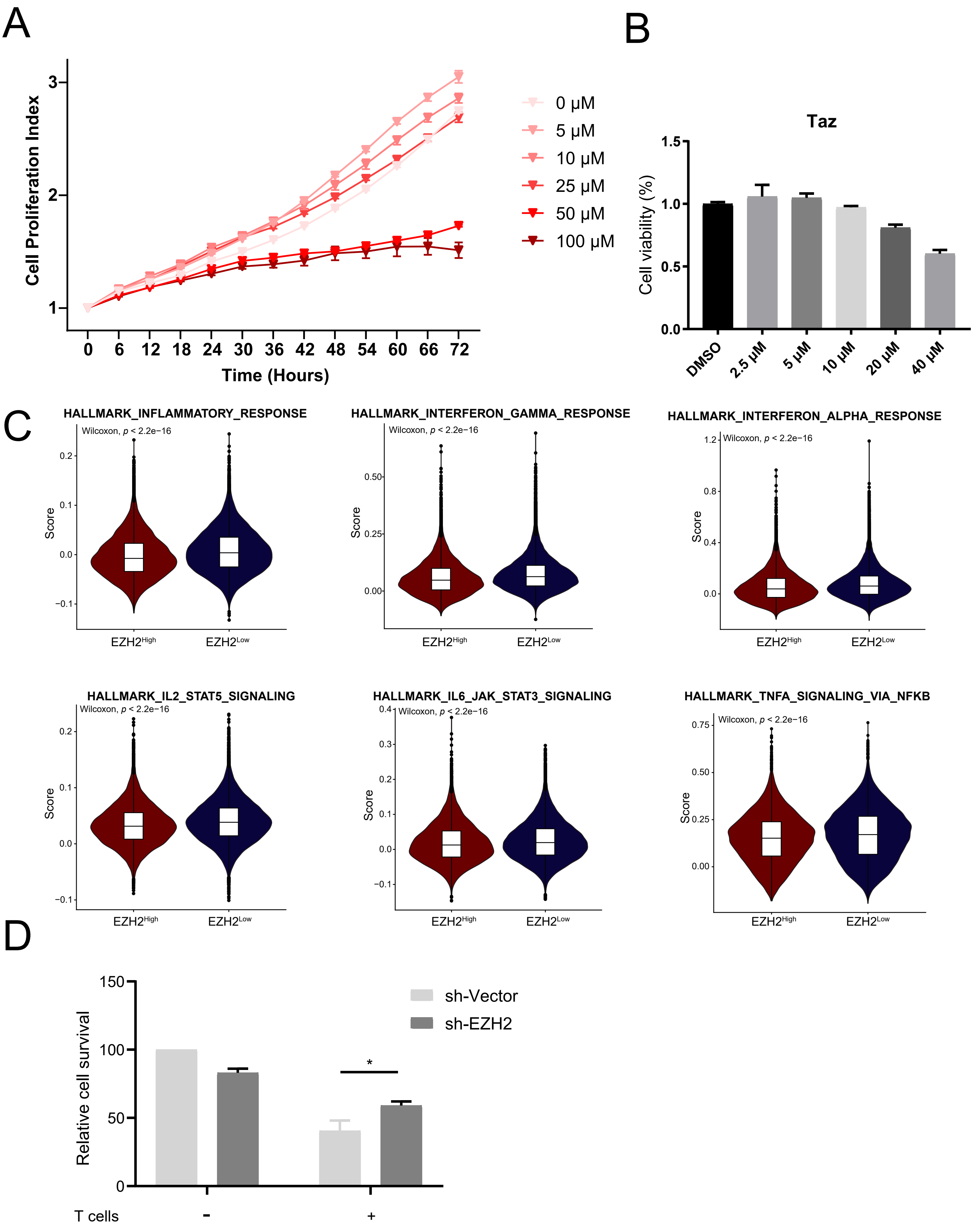

A
Cell Proliferation Index
Time (Hours)
0 μM
5 μM
10 μM
25 μM
50 μM
100 μM
B
Taz
Cell viability (%)
DMSO
2.5 μM
5 μM
10 μM
20 μM
40 μM
C
HALLMARK_INFLAMMATORY_RESPONSE
HALLMARK_INTERFERON_GAMMA_RESPONSE
HALLMARK_INTERFERON_ALPHA_RESPONSE
HALLMARK_IL2_STAT5_SIGNALING
HALLMARK_IL6_JAK_STAT3_SIGNALING
HALLMARK_TNFA_SIGNALING_VIA_NFKB
Wilcoxon, p < 2.2e−16
Score
EZH2High
EZH2Low
D
sh-Vector
sh-EZH2
Relative cell survival
T cells
-
+
*

Figure S2

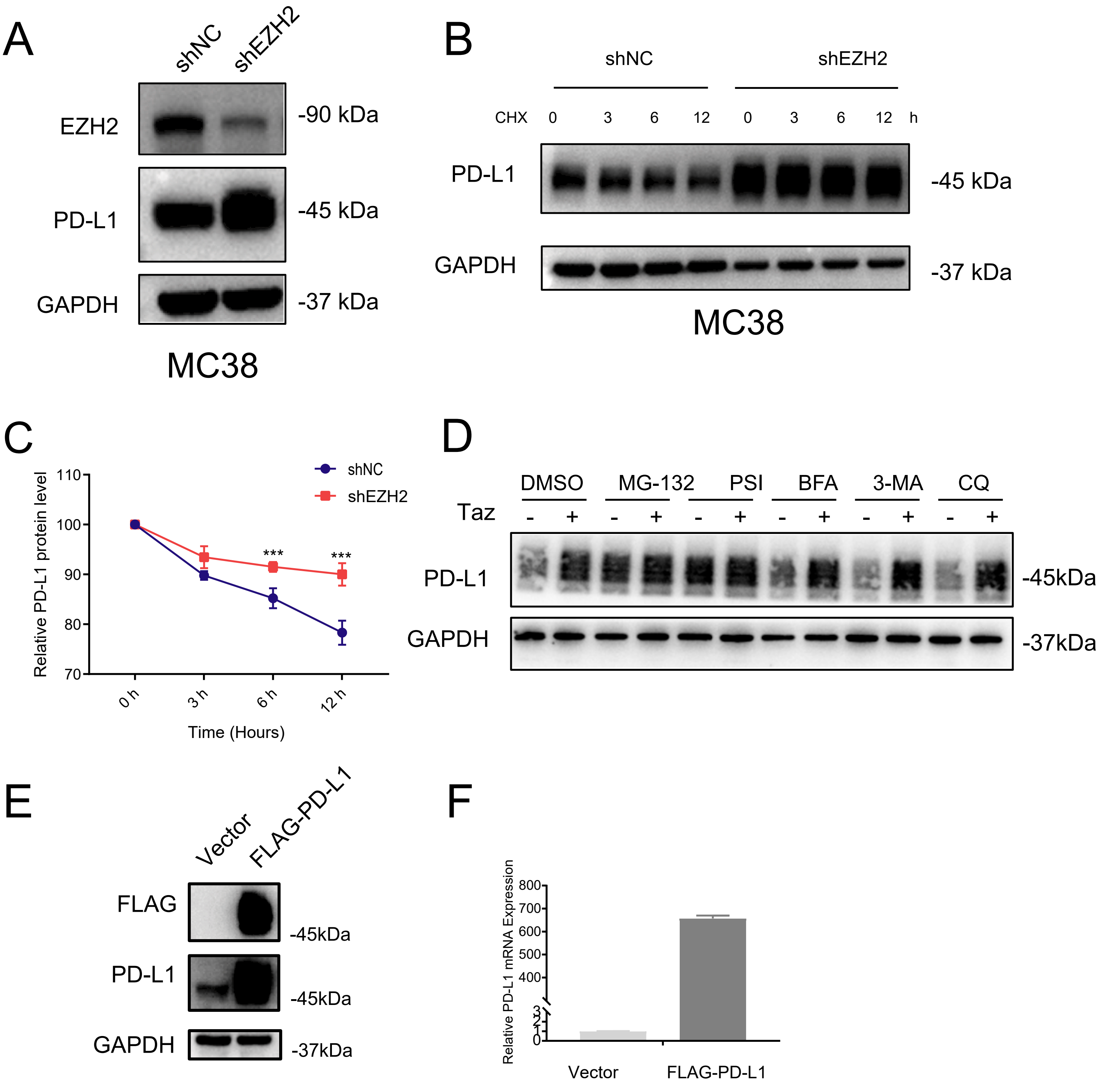
A
shNC
shEZH2
EZH2
-90 kDa
PD-L1
-45 kDa
GAPDH
-37 kDa
MC38
B
shNC
shEZH2
CHX 0 3 6 12 0 3 6 12 h
PD-L1
-45 kDa
GAPDH
-37 kDa
MC38
C
shNC
shEZH2
Relative PD-L1 protein level
110
100
90
80
70
***
***
0 h
3 h
6 h
12 h
Time (Hours)
D
DMSO
MG-132
PSI
BFA
3-MA
CQ
Taz - + - + - + - + - + - +
PD-L1
-45kDa
GAPDH
-37kDa
E
Vector
FLAG-PD-L1
FLAG
-45kDa
PD-L1
-45kDa
GAPDH
-37kDa
F
Relative PD-L1 mRNA Expression
800
700
600
500
400
3
2
1
0
Vector
FLAG-PD-L1

Figure S3

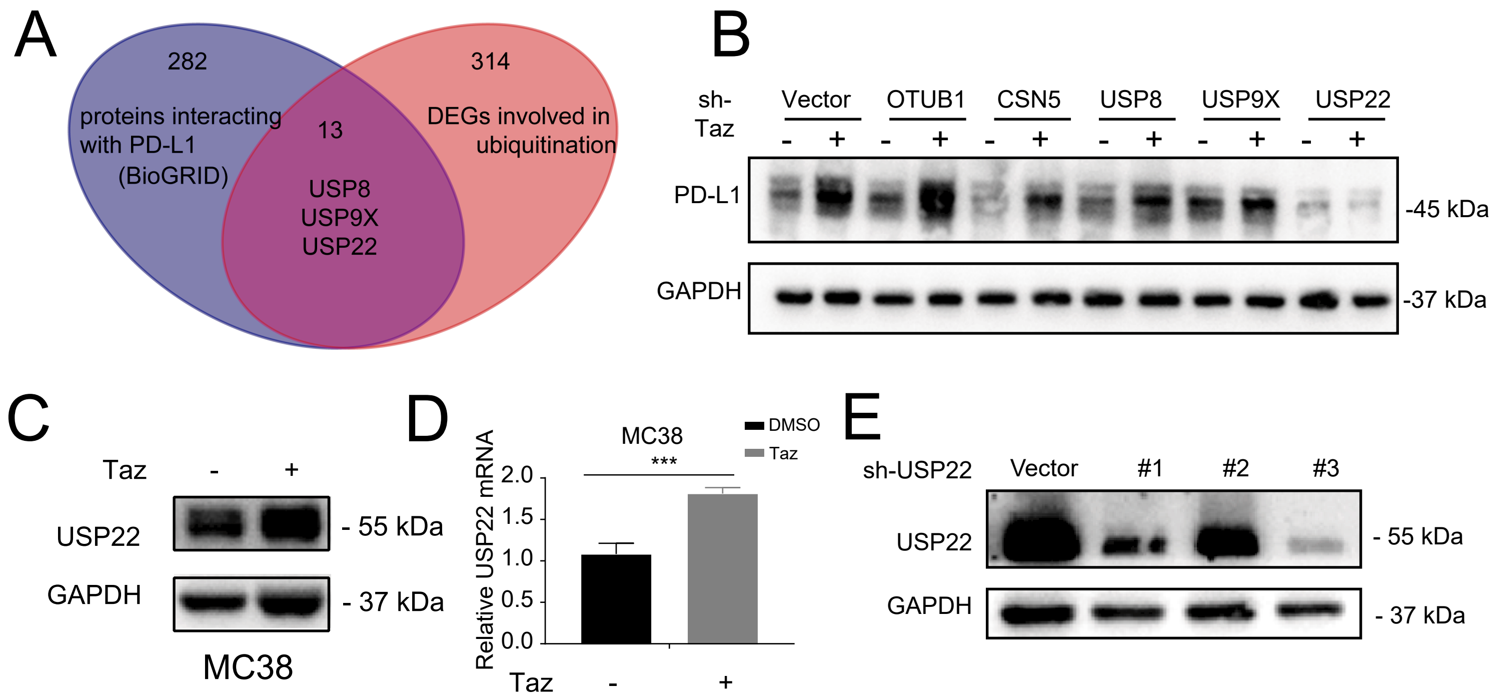

A
282
proteins interacting with PD-L1 (BioGRID)
13
USP8
USP9X
USP22
314
DEGs involved in ubiquitination
B
sh-
Taz
Vector
OTUB1
CSN5
USP8
USP9X
USP22
PD-L1
-45 kDa
GAPDH
-37 kDa
C
Taz
USP22
- 55 kDa
GAPDH
- 37 kDa
MC38
D
MC38
***
DMSO
Taz
Relative USP22 mRNA
2.0
1.5
1.0
0.5
0.0
E
sh-USP22
Vector
#1
#2
#3
USP22
- 55 kDa
GAPDH
- 37 kDa

Figure S4

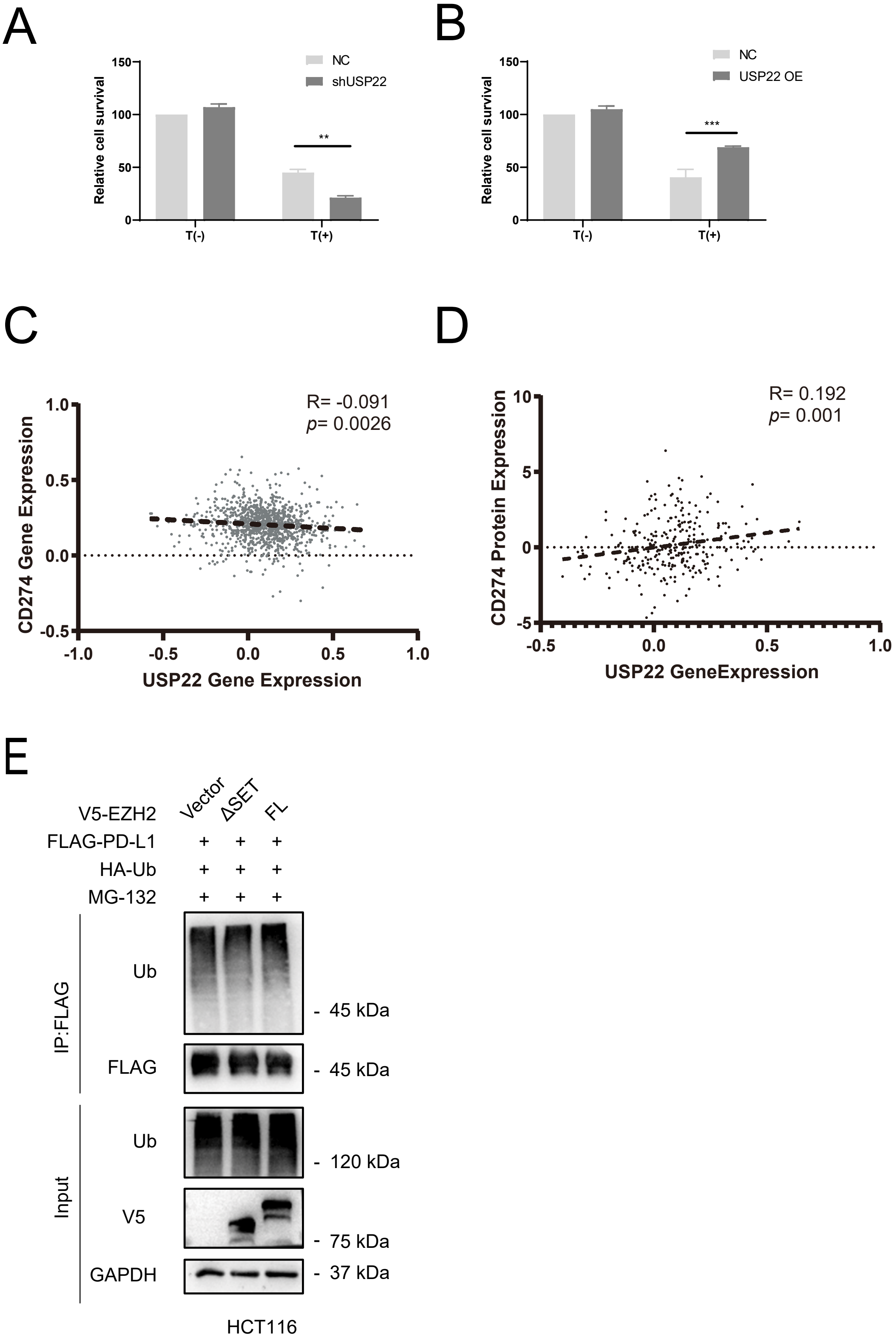

A
Relative cell survival
150
100
50
0
NC
shUSP22
**
T(-)
T(+)
B
Relative cell survival
150
100
50
0
NC
USP22 OE
***
T(-)
T(+)
C
R= -0.091
p= 0.0026
CD274 Gene Expression
1.0
0.5
0.0
-0.5
-1.0
-0.5
0.0
0.5
1.0
USP22 Gene Expression
D
R= 0.192
p= 0.001
CD274 Protein Expression
10
5
0
-5
-0.5
0.0
0.5
1.0
USP22 GeneExpression
E
V5-EZH2
Vector
ΔSET
FL
FLAG-PD-L1
HA-Ub
MG-132
+ + +
IP:FLAG
Ub
- 45 kDa
FLAG
- 45 kDa
Input
Ub
- 120 kDa
V5
- 75 kDa
GAPDH
- 37 kDa
HCT116

Figure S5

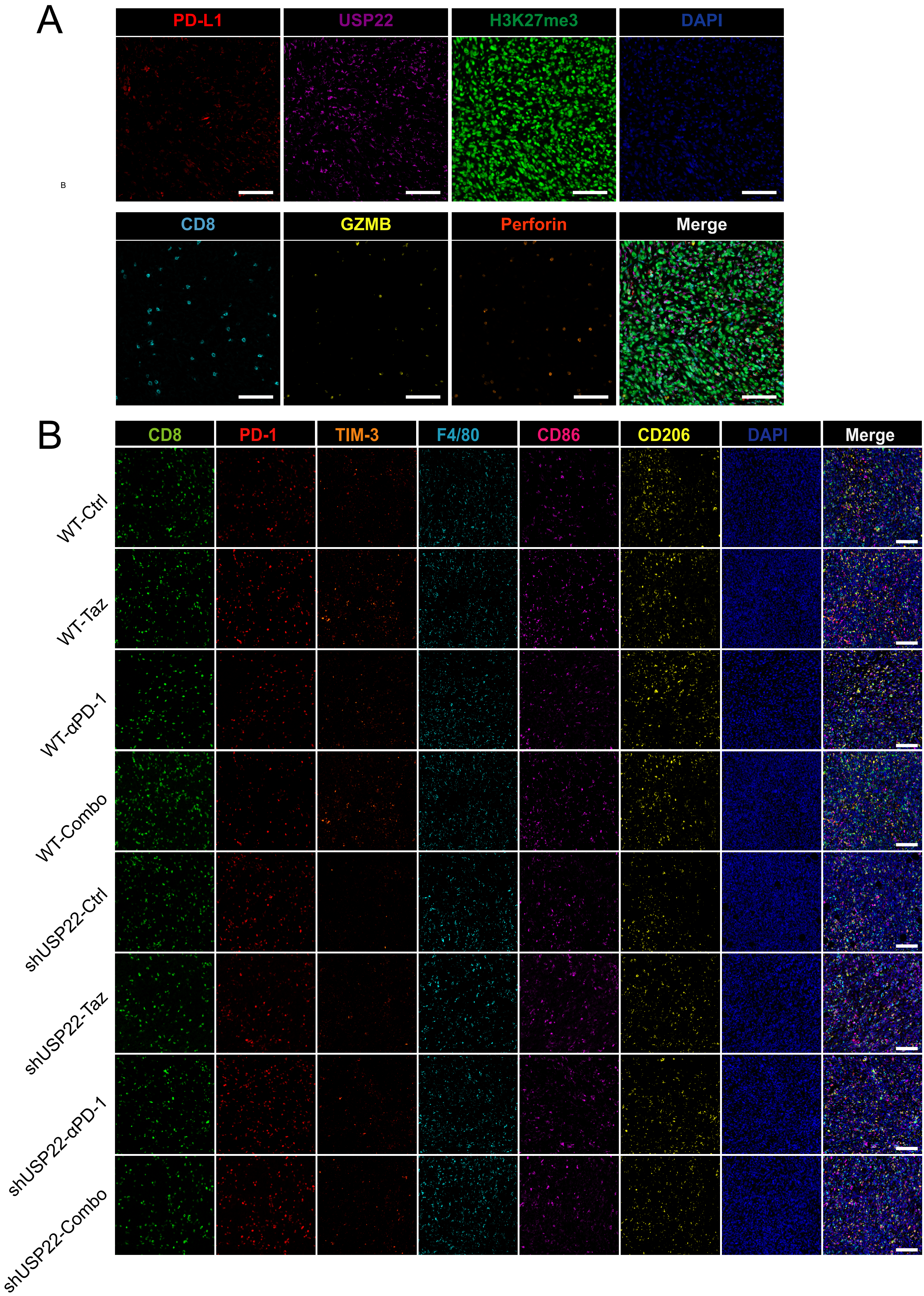
A
PD-L1
USP22
H3K27me3
DAPI
B
CD8
GZMB
Perforin
Merge
B
CD8
PD-1
TIM-3
F4/80
CD86
CD206
DAPI
Merge
WT-Ctrl
WT-Taz
WT-αPD-1
WT-Combo
shUSP22-Ctrl
shUSP22-Taz
shUSP22-αPD-1
shUSP22-Combo

Figure S6

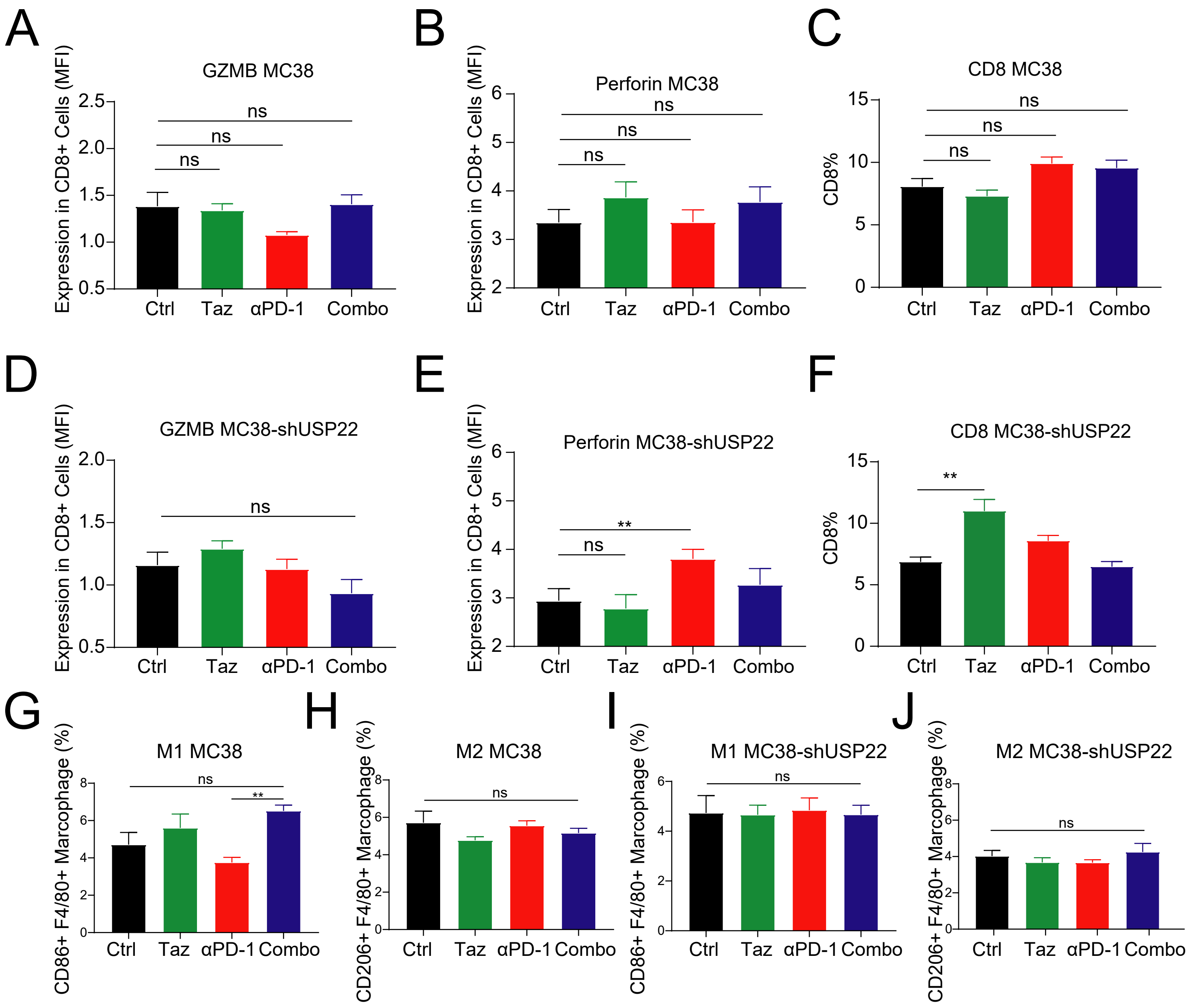
A
GZMB MC38
Expression in CD8+ Cells (MFI)
ns
ns
ns
Ctrl
Taz
αPD-1
Combo
B
Perforin MC38
Expression in CD8+ Cells (MFI)
ns
ns
ns
C
CD8 MC38
CD8%
ns
ns
ns
D
GZMB MC38-shUSP22
ns
E
Perforin MC38-shUSP22
**
ns
F
CD8 MC38-shUSP22
**
G
M1 MC38
CD86+ F4/80+ Marcophage (%)
ns
**
H
M2 MC38
CD206+ F4/80+ Marcophage (%)
ns
I
M1 MC38-shUSP22
CD86+ F4/80+ Marcophage (%)
ns
J
M2 MC38-shUSP22
CD206+ F4/80+ Marcophage (%)
ns

Figure S7

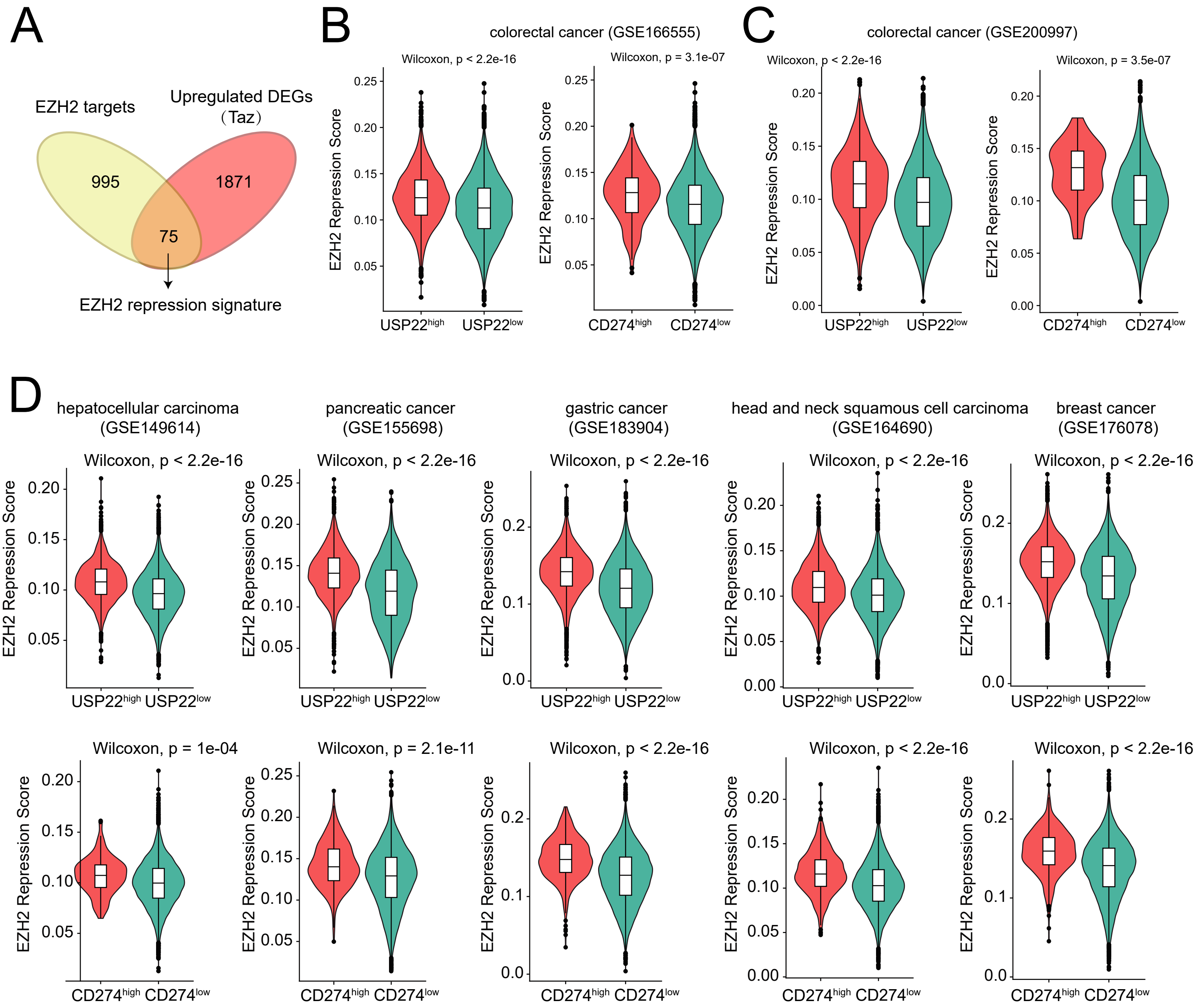
A
EZH2 targets
Upregulated DEGs (Taz)
995
1871
75
EZH2 repression signature
B
colorectal cancer (GSE166555)
Wilcoxon, p < 2.2e-16
Wilcoxon, p = 3.1e-07
EZH2 Repression Score
$USP22^{high}$ $USP22^{low}$
$CD274^{high}$ $CD274^{low}$
C
colorectal cancer (GSE200997)
Wilcoxon, p < 2.2e-16
Wilcoxon, p = 3.5e-07
D
hepatocellular carcinoma (GSE149614)
pancreatic cancer (GSE155698)
gastric cancer (GSE183904)
head and neck squamous cell carcinoma (GSE164690)
breast cancer (GSE176078)
Wilcoxon, p < 2.2e-16
Wilcoxon, p = 1e-04
Wilcoxon, p = 2.1e-11

Figure S8

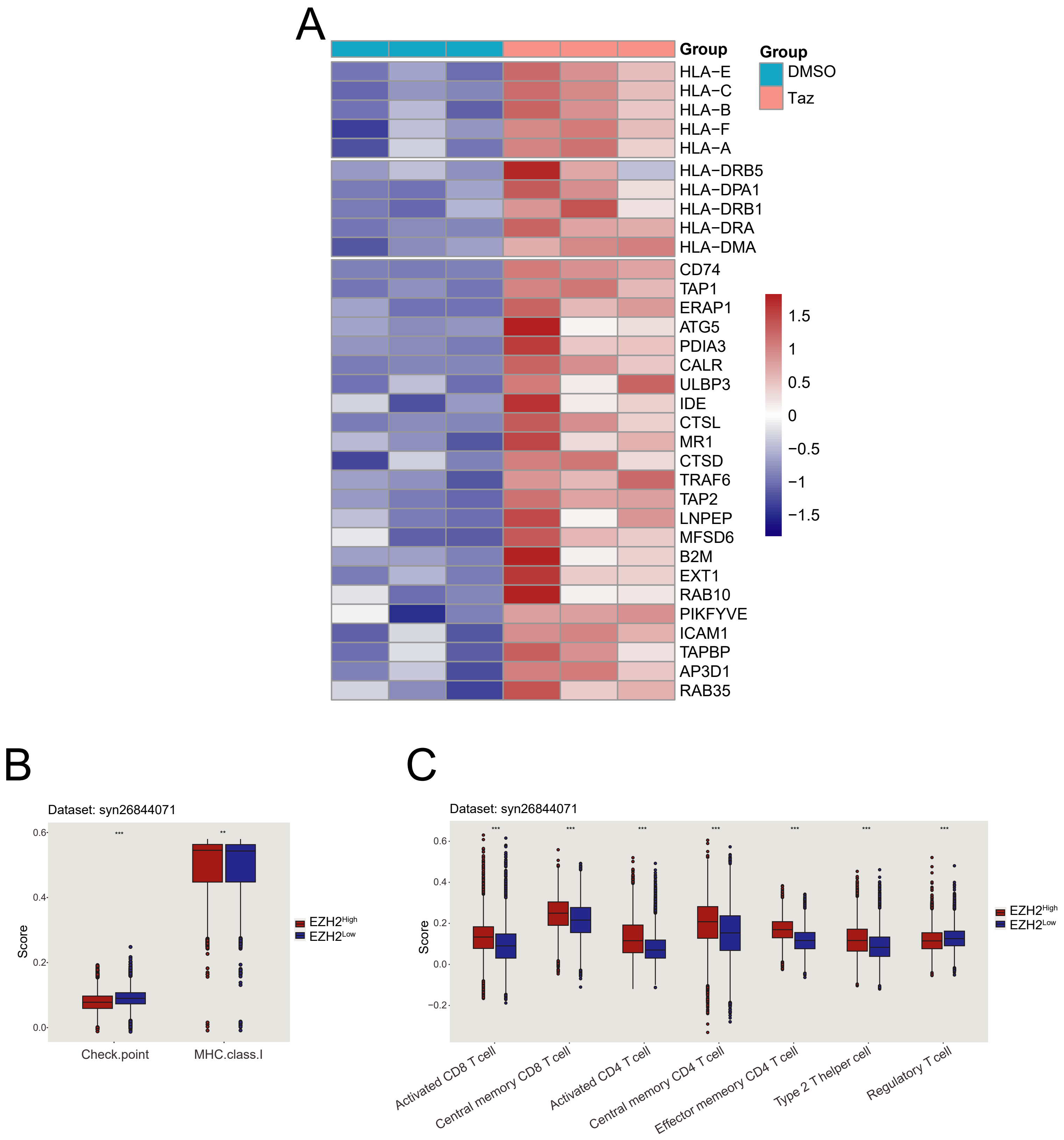
A
Group
Group
DMSO
Taz
HLA−E
HLA−C
HLA−B
HLA−F
HLA−A
HLA−DRB5
HLA−DPA1
HLA−DRB1
HLA−DRA
HLA−DMA
CD74
TAP1
ERAP1
ATG5
PDIA3
CALR
ULBP3
IDE
CTSL
MR1
CTSD
TRAF6
TAP2
LNPEP
MFSD6
B2M
EXT1
RAB10
PIKFYVE
ICAM1
TAPBP
AP3D1
RAB35
1.5
1
0.5
0
−0.5
−1
−1.5
B
Dataset: syn26844071
Score
EZH2High
EZH2Low
Check.point
MHC.class.I
C
Dataset: syn26844071
Score
EZH2High
EZH2Low
Activated CD8 T cell
Central memory CD8 T cell
Activated CD4 T cell
Central memory CD4 T cell
Effector memeory CD4 T cell
Type 2 Thelper cell
Regulatory T cell
